# Genomic Analysis of the Kiwifruit Pathogen *Pseudomonas syringae* pv. *actinidiae* Provides Insight into the Origins of an Emergent Plant Disease

**DOI:** 10.1371/journal.ppat.1003503

**Published:** 2013-07-25

**Authors:** Honour C. McCann, Erik H. A. Rikkerink, Frederic Bertels, Mark Fiers, Ashley Lu, Jonathan Rees-George, Mark T. Andersen, Andrew P. Gleave, Bernhard Haubold, Mark W. Wohlers, David S. Guttman, Pauline W. Wang, Christina Straub, Joel Vanneste, Paul B. Rainey, Matthew D. Templeton

**Affiliations:** 1 New Zealand Institute for Advanced Study and Allan Wilson Centre, Massey University, Auckland, New Zealand; 2 Centre for the Analysis of Genome Evolution and Function, University of Toronto, Toronto, Canada; 3 The New Zealand Institute for Plant and Food Research Limited, Auckland, New Zealand; 4 Biozentrum, University of Basel and Swiss Institute of Bioinformatics, Basel, Switzerland; 5 The New Zealand Institute for Plant and Food Research Limited, Lincoln, New Zealand; 6 Max Planck Institute for Evolutionary Biology, Plön, Germany; 7 The New Zealand Institute for Plant and Food Research Limited, Ruakura, Hamilton, New Zealand; 8 School of Biological Sciences, University of Auckland, Auckland, New Zealand; The University of North Carolina at Chapel Hill, United States of America

## Abstract

The origins of crop diseases are linked to domestication of plants. Most crops were domesticated centuries – even millennia – ago, thus limiting opportunity to understand the concomitant emergence of disease. Kiwifruit (*Actinidia* spp.) is an exception: domestication began in the 1930s with outbreaks of canker disease caused by *P. syringae* pv. *actinidiae* (*Psa*) first recorded in the 1980s. Based on SNP analyses of two circularized and 34 draft genomes, we show that *Psa* is comprised of distinct clades exhibiting negligible within-clade diversity, consistent with disease arising by independent samplings from a source population. Three clades correspond to their geographical source of isolation; a fourth, encompassing the *Psa*-V lineage responsible for the 2008 outbreak, is now globally distributed. *Psa* has an overall clonal population structure, however, genomes carry a marked signature of within-pathovar recombination. SNP analysis of *Psa*-V reveals hundreds of polymorphisms; however, most reside within PPHGI-1-like conjugative elements whose evolution is unlinked to the core genome. Removal of SNPs due to recombination yields an uninformative (star-like) phylogeny consistent with diversification of *Psa*-V from a single clone within the last ten years. Growth assays provide evidence of cultivar specificity, with rapid systemic movement of *Psa*-V in *Actinidia chinensis*. Genomic comparisons show a dynamic genome with evidence of positive selection on type III effectors and other candidate virulence genes. Each clade has highly varied complements of accessory genes encoding effectors and toxins with evidence of gain and loss via multiple genetic routes. Genes with orthologs in vascular pathogens were found exclusively within *Psa*-V. Our analyses capture a pathogen in the early stages of emergence from a predicted source population associated with wild *Actinidia* species. In addition to candidate genes as targets for resistance breeding programs, our findings highlight the importance of the source population as a reservoir of new disease.

## Author Summary

Despite considerable scientific advances in plant protection during the last century, agricultural crops remain vulnerable to infection by pathogens. The intensive cultivation particularly of clonally propagated crop plants increases the potential for the emergence and rapid spread of new diseases. *Pseudomonas syringae* pv. *actinidiae* was first reported as a canker-causing pathogen of kiwifruit in the mid-1980s. However, a new outbreak of the disease occurred in 2008 and this strain has spread rapidly throughout growing regions of the world. In order to determine the origin, population structure and defining features of this pathogen, a large-scale sequencing project was established. This clarified the phylogenetic relationships between the different *Psa* isolates and identified the outbreak-specific gene sets associated with the aggressive systemic infection strategy exhibited by the virulent strain. This information is invaluable in developing robust long-term solutions for this serious disease. Given that kiwifruit production on a commercial scale is a relatively recent event, this analysis provides a unique insight into the evolution of this pathogen with its host, from its first emergence to the latest global outbreak. This understanding should aid in the mitigation of devastating outbreaks in the future.

## Introduction

Despite considerable improvements in the management of plant diseases, modern agriculture remains vulnerable to losses caused by microbial pathogens. Plant diseases conservatively account for the loss of at least 10% of annual global food production [1]. The intensive cultivation of clonally propagated plants with low genetic diversity heightens opportunities for the emergence and rapid spread of infectious disease [1]–[3].

The origins of agricultural plant diseases are unclear [3], [4]. The earliest pathogens are likely to have evolved from commensals or pathogens colonizing wild relatives of plants selected for human domestication [5]–[10]. Given that domestication of staple crop plants took place centuries (and often millennia) ago, the signal of this evolutionary past – the nature of the initial pathogen population, its relationship with commensal types, its diversity and genetic structure, plus factors and processes that might have led to the first outbreaks of disease – is obscured by the passage of time. Kiwifruit (*Actinidia* spp.) is an exception. Domestication of kiwifruit is recent and clearly documented; outbreaks of disease are recorded and the pathogens responsible have been preserved.

The genus *Actinidia* comprises 55 species and about 76 taxa native to eastern Asia, with the greatest abundance and diversity in the southwestern provinces of China [11]. Fruit have long been collected from the wild, yet commercial cultivation only began to gain momentum in the 1980s, based on the success of *A. deliciosa* ‘Hayward’ developed in New Zealand during the 1930s. Subsequently cultivars from the species *A. chinensis*, such as ‘Hort16A’, ‘Jin Tao’ and ‘Hongyang’ were commercialized in several kiwifruit growing regions, including New Zealand, China (1998), Italy (2001) and Chile (2003) [12].

A small group of fungal and bacterial diseases on vines, roots and fruit of ‘Hayward’ were recognized once commercial plantings became substantial in the 1980s [13]. Kiwifruit canker disease caused by *Pseudomonas syringae* pv. *actinidiae* (*Psa*) was first reported and characterized on *A. deliciosa* in Shizuoka, Japan in 1984 [14]; in that same year bacterial canker disease was also reported in an orchard in Hunan, China [15]. Canker disease was subsequently observed in Korea (1988) and Italy (1992) [16], [17]. Symptoms of infection include late winter die-back of young canes, frequently accompanied by rust red exudates from canes and trunks, and the presence of necrotic lesions with chlorotic halos on leaves during the spring [18], [19].

In 2008 an aggressive form of *Psa* was reported in Italy on *A. chinensis*. Multi-locus sequencing showed the pathogenic strains to be divergent from earlier Italian and Japanese isolates [20]–[23]). The virulent form of *Psa* was subsequently detected in neighboring European countries [24], China, Chile and New Zealand [23], [25]. Additional genomic analyses confirmed the clonal nature of the disease outbreak and its distinctive genetic composition [19], [22], [26], [27]. Throughout the paper we refer to this recent epidemic as the “2008 outbreak” with the strains responsible being referred to collectively as “*Psa*-V”.

Rapid transmission and increased severity of infection arising from the 2008 outbreak has had devastating effects leading to complete destruction of orchards. In 2010, *Psa*-V was detected in New Zealand, where kiwifruit is the most valuable horticultural export [12], [25]. *Psa*-V spread rapidly from its initial incursion site in the Bay of Plenty. Within two years the number of infected orchards rose from three to 1232 (37% of New Zealand orchards) and continues to increase [28].

Draft genome sequencing of one Italian isolate from the 2008 outbreak, plus another strain from an epidemic in 1992, showed the earlier Italian strain to be identical to a 1984 Japanese isolate, but different to the 2008 (*Psa*-V) outbreak strains, particularly with respect to effector inventories [26]. Draft genomes of four additional isolates from the 2008 Italian outbreak plus three isolates from China and two from Chile also belong to the same *Psa*-V lineage although one Chinese strain (M228) appears divergent [22], [27]. Strains of the *Psa*-V lineage were shown to differ by as few as six SNPs. A genomic island with similarity to PPHGI from *Pseudomonas syringae* pv. *phaseolicola* (*Pph*) was characterized and shown to differ between the European, Chilean and Chinese/New Zealand isolates [22], [27], [29]. The full extent of the genetic distinctiveness of *Psa*-V relative to strains from early outbreaks remains uncertain.

While work to date shows that the 2008 epidemic is caused by a strain distinct from previous outbreaks of canker disease, it is not clear whether *Psa*-V evolved from earlier outbreaks or whether it has independent origins. Clarification can come from studies that determine patterns of nucleotide diversity from strains sampled from different disease outbreaks, at different time points, from different geographical locations [30]–[33]. From the analysis of polymorphism data it becomes possible to infer underlying population processes and, provided recombination is not extensive, phylogeny [34], [35]. Leaving aside opportunities for insight into the evolution of a newly emergent pathogen, knowledge of population structure also has important implications for the development of strategies for disease control, including protocols to prevent future outbreaks.

Here we report an in depth analysis of *Psa* evolution based upon complete genome sequences of a *Psa*-V strain from the New Zealand outbreak and the type strain (J-35, ICMP9617) originally isolated in Japan, plus 34 additional draft genomes that encompass strains from previously known outbreaks. Our analyses provide evidence of a single source population from which outbreaks of disease have arisen *via* independent transmission events to different kiwifruit growing regions. Overall the population is clonal, however, approximately 10% of the genome shows evidence of homologous recombination marked by gene conversion. Comparative analyses reveal dynamic genomes with positive selection affecting type III effectors and candidate virulence genes with *Psa*-V containing numerous genes found in pathovars of vascular plants. *In planta* growth data provide a link between genomic inferences and ecological performance on host cultivars. Together these data provide understanding of the processes and factors affecting the emergence of a new plant pathogen.

## Materials and Methods

### Isolates

The 25 strains of *P. syringae* pv. *actinidiae* were isolated from *Actinidia* spp. in Japan, Korea, Italy and New Zealand. Details of dates and locations of isolations are in Table 1. Bacteria were maintained on King's B (KB) agar plates [36] and stored at −80°C in glycerol. Where possible, cultures have been deposited in the International Collection of Micro-organisms from Plants (ICMP; www.landcareresearch.co.nz). Several isolates were sourced from overseas collections, including the Korean Agricultural Culture Collection (KACC), Suwon, Republic of Korea, the National Institute of Agrobiological Sciences (NIAS), 2-1-2 Kannondai, Tsukuba, Ibaraki 305-8602, Japan, the National Collection of Plant Pathogenic Bacteria (NCPPB), Food and Environment Research Agency, Sand Hutton, York, UK.

**Table 1:** Strain table and assembly statistics.

Isolate ID	WGS origin	Host plant	Country	Year	ICMP number	Other collection/alias	Genbank accession	Contigs^2^	N50	Longest scaffold
*Psa* C-1	Mazzaglia *et al.* (2012)	*A. chinensis* ‘Hongyang’	China, Shaanxi	2010		CH2010-6	AGUH	*342*	51,971	198,200
*Psa* C-9	Butler *et al.* 2013		China, Shaanxi	2010		M228	ANJI	3259	4561	57,016
*Psa* Cl-4	Butler *et al.* 2013	*A. deliciosa*	Chile, Maule	2010	19439	Psa1B	ANJM	477	36,822	142,020
*Psa* Cl-5	Butler *et al.* 2013	*A. deliciosa*	Chile, Maule	2010	19455	286532	ANJK	415	39,400	127,854
*Psa* I-1	Marceletti *et al.* (2011)	*A. deliciosa* ‘Hayward’	Italy	1992		NCPPB 3871/I-Psa	AFTF	466	27,730	122,209
*Psa* I-10	Butler *et al.* 2013	*A. deliciosa*	Italy, Rome	2010	18744	CRA-FRU 11.41	ANGD	422	35,308	143,214
*Psa* I-12	This study	*A. chinensis* ‘Hort16A’	Italy, Latina	2010	19079	I.27.4.10	AOKL	3,045	4,064	57,811
*Psa* I-2	Marceletti *et al.* (2011)	*A. chinensis* ‘Hort16A’	Italy	2008		CRAFRU8.43/I2-Psa	AFTG	590	22,372	85,982
*Psa* I-3	Mazzaglia *et al.* (2012)	*A. chinensis* ‘Hort16A’	Italy, Lazio	2008		CFBP 7286	AGNO	*352*	43,501	139,438
*Psa* J-1	Baltrus *et al.* (2011)	*A. deliciosa*	Japan	1984		MAFF 302091	AEAL	*138*	69,188	N/A
*Psa* J-2	Mazzaglia *et al.* (2012)	*A. chinensis*	Japan	1988		PA459	AGNQ	*393*	49,861	303,211
*Psa* J-25	Mazzaglia *et al.* (2012)	*A. deliciosa* ‘Hayward’	Japan, Shizuoka	1984	9855	KW41	AGNP	*429*	38,609	136,008
*Psa* J-29	This study	*A. arguta*	Japan, Kanagawa	1987	19102	MAFF 302133/JpSar1	AOKA	548	27,853	113,869
*Psa* J-30	This study	*A. arguta*	Japan, Kanagawa	1987	19103	MAFF 302134/JpSar2	AOJQ	527	30,580	145,811
*Psa* J-31	This study	*A. deliciosa* ‘Hayward’	Japan, Kanagawa	1987	19070	MAFF 302143/JpKiw4	AOJY	2,844	3,960	27,127
*Psa* J-32	This study	*A. deliciosa* ‘Hayward’	Japan, Wakayama	1988	19068	MAFF 302145/JpWa1	AOJX	790	35,303	133,336
*Psa* J-33	This study	*A. deliciosa* ‘Hayward’	Japan, Wakayama	1988	19104	MAFF 302146/JpWa2	AOJZ	496	34,688	133,960
*Psa* J-35	This study	*A. deliciosa* ‘Hayward’	Japan, Shizuoka	1984	9617	NCPPB 3739/Kw11	AOKP^3^	*2*	158,837	6,466,847
*Psa* K-26	This study	*A. chinensis*	Korea, Jeonnam	1997	19072	KACC 10584/Kr26	AOJW	350	43,039	169,668
*Psa* K-27	This study	*A. chinensis*	Korea, Jeonnam	1998	19073	KACC 10594/Kr27	AOJR	926	17,194	105,639
*Psa* K-28	This study	*A. chinensis*	Korea, Jeonnam	1997	19071	KACC 10754/Kr28	AOJS	459	29,527	107,707
*Psa* NZ LV-11	This study	*A. chinensis* ‘Hort16A’	New Zealand, Bay of Plenty	2010	18804		AOJU	4,811	1,704	34,764
*Psa* NZ LV-14	This study	*A. deliciosa* ‘Hayward’	New Zealand, Tauranga	2010	18807		AOKG	3,227	3,876	38,937
*Psa* NZ LV-17	This study	*A. chinensis* ‘Hort16A’	New Zealand, Katikati	2010	19096		AOKF	4,832	2,333	19,396
*Psa* NZ LV-18	This study	*A. chinensis* ‘Hort16A’	New Zealand, Motueka	2010	19098		AOKE	391	49,576	136,769
*Psa* NZ LV-19	This study	*A. chinensis* ‘Hort16A’	New Zealand, Te Puke	2010	19099		AOKD	417	43,791	164,509
*Psa* NZ LV-20	This study	*A. chinensis* ‘Hort16A’	New Zealand, Te Puke	2010	19100		AOKC	631	26,586	113,072
*Psa* NZ LV-5	This study	*A. chinensis* ‘Hort16A’	New Zealand, Hawke's Bay	2010	18803		AOKK	351	55,333	144,243
*Psa* NZ LV-6	This study	*A. chinensis* ‘Hort16A’	New Zealand, Te Puke	2010	19094		AOKJ	586	26,883	111,577
*Psa* NZ LV-8	This study	*A. chinensis* ‘Hort16A’	New Zealand, Te Puke	2010	19095		AOKI	849	15,642	70,310
*Psa* NZ LV-9	This study	*A. deliciosa* ‘Hayward’	New Zealand, Golden Bay	2010	18883		AOKH	502	34,879	147,278
*Psa* NZ V-1	This study	*A. deliciosa* ‘Hayward’	New Zealand, Te Puke	2010	18886		AOJT	2,016	6,501	53,135
*Psa* NZ V-13	This study	*A. deliciosa* ‘Hayward’	New Zealand, Te Puke	2010	18884		AOKO	*2*	163,512	6,504,601
*Psa* NZ V-15	This study	*A. deliciosa* ‘Hayward’	New Zealand, Te Puke	2010	19101		AOKM	2,171	6,108	73,690
*Psa* NZ V-16	This study	*A. chinensis* ‘Hort16A’	New Zealand, Te Puke	2010	18801		AOKQ	800	23,499	100,404
*Psa* NZ V-23	This study	*A. chinensis* ‘Hort16A’	New Zealand, Te Puke	2010	19097	10787	AOKN	843	20,872	80,234
*Pmp* M302280	Baltrus *et al.* (2011)	*Prunus* spp.	England	1931		MAFF 302280	AEAE	969	15161	N/A
*Pmp*	N/A^1^	*Prunus avium*	New Zealand, Alexandra	1972	3676			N/A	N/A	N/A
*Pth* 2598	Mazzaglia *et al.* (2012)	*Camellia sinensis*	Japan	1970	3923	NCPPB 2598	AGNN	*218*	7937	242,733
*Pto* DC3000	Buell *et al.* (2003)	*Solanum lycopersicon*	England	1960	2844	NCPPB 1106	AE016853	2	N/A	6,397,126

1 A New Zealand *Pmp* isolate was used solely for the pathogenicity assay.

2 Italicized contig entries refer to the number of scaffolds in the assembly. Various assembly programs were used by different authors so these data are not directly comparable.

3 A prior version of NCPPB3739 was deposited in NCBI with the Genbank accession AFTH.

### DNA extraction

Cultures were grown overnight in KB or nutrient broth. DNA for whole genome sequencing was purified using the PureGene DNA Isolation kit (Qiagen, Hilden, Germany) with some modifications. For each 750 µL of culture extracted, reagent volumes were doubled and the protein precipitation step was carried out twice. Purified DNA was quantified and its purity assessed using a nanodrop spectrophotometer (NanoDrop Technologies, Rockland, DE).

### Genome sequencing and assembly

Paired-end libraries were generated from genomic DNA and sequenced using the Illumina GAII instrument at the Center for Genome Analysis and Function (CAGEF, Toronto). The raw data were filtered using fastq-mcf (https://code.google.com/p/ea-utils/wiki/FastqMcf) and quality checked using fastqc (http://www.bioinformatics.babraham.ac.uk/projects/fastqc/). Contigs were built using the SOAP *de novo* assembler (http://soap.genomics.org.cn/soapdenovo.html) with a k-mer value of 37. Sequencing and assembly statistics for each strain are shown in Table 1. Additional sequencing was performed on two *Psa* isolates, NZ V-13 (a virulent (*Psa*-V) strain isolated during the 2010 outbreak in New Zealand), and the *Psa* type strain (J-35, ICMP9617 originally isolated in 1984 from *Actinidia* in Japan) using a 10 kb mate-end library on the Roche 454 platform by Macrogen, Korea (www.macrogen.com). Scaffolds were generated using the Roche Newbler assembler, and resulted in 14 and 12 scaffolds greater than 2 kb, for NZ V-13 and J-35, respectively. The quality of all assemblies was improved by iteratively filling in short tracts of Ns using GapCloser (version 1.12, http://soap.genomics.org.cn/soapdenovo.html) and scaffolding with the mate-pair data using SSPACE [37]. The two Newbler assemblies were used as a basis for generating high quality reference sequences by manually integrating the Illumina-based contigs using Geneious (version 5.6.3, Biomatters, http://www.geneious.com/). The remaining large scaffolds were linked by designing primers 1–2 kb from the 5′ and 3′ ends and using long-template PCR (Takara Bio inc., Shiga, Japan) with amplification conditions recommended by the manufacturer. The quality of the genome assemblies was visually inspected using Hagfish (https://github.com/mfiers/hagfish/) to align paired-end reads back to the draft genome. Misaligned and dubious contigs were manually filtered. Additional gap-filling was performed on *Psa* NZ V-13 with primers flanking each gap. Products were purified and Sanger sequenced (Macrogen, Korea, www.macrogen.com). Assemblies for all strains were submitted to the Prokaryotic Genomes Automatic Annotation Pipeline (PGAAP, https://www.ncbi.nlm.nih.gov/genomes/static/Pipeline.html) for gene prediction and annotation: they are available from GenBank (Table 1).

### Plasmid preparation and Pulsed-Field Gel Electrophoresis analysis

Plasmid DNA was extracted from 250 mL cultures using an alkaline lysis with SDS extraction method (Protocol 3: Preparation of Plasmid DNA by Alkaline Lysis with SDS: Maxipreparation) [38]. Nutrient Broth (250 mL) was inoculated with a 2 mL overnight culture and left shaking for 48 h at 28°C. Cells were harvested by centrifugation at 12,000 *g* and either used fresh or stored at −20°C until required. Cells were resuspended in 18 mL Alkaline Lysis Solution I (50 mM Glucose, 25 mM Tris (pH 8.0), 10 mM EDTA (pH 8.0)), and then 2 mL freshly prepared Lysozyme solution (10 mg/mL) was added. Two volumes (40 mL) of freshly prepared Alkaline Lysis Solution II (0.2 M NaOH, 1% SDS) were added, the cells mixed by gentle inversion several times, and then incubated at room temperature (RT). After 5–10 min of incubation, 20 mL ice-cold Alkaline Lysis Solution III (5 M KAcetate 60 mL, glacial acetic acid, 11.5 mL H_2_O, 28.5 mL) was added, and contents mixed by gentle but effective swirling. Samples were incubated on ice for 10 min and then centrifuged at 20,000 *g* for 30 min at 5°C. The supernatant was separated from the pellet, the volume measured, 0.6 vol of isopropanol was added and samples were incubated for 10 min at RT. After centrifugation at 12,000 *g*, the supernatant was discarded, and the pellet washed in 70% ethanol, drained and left to dry (without vacuum) for 5–10 min at RT. The pellet was resuspended in 500 µL Tris/EDTA (pH 8.0). Concentration of samples was achieved by further ethanol precipitation.

Plasmid digests were run using a Chef-DR III system (Bio-Rad, Hercules CA). Volumes of up to 20 µL were loaded onto 1.0% 1×TAE agarose gels and run in 1×TAE buffer for 18 h at 15°C at 6 V/cm. The switch time was 5–15 s with an angle of 120°. Gels were stained with ethidium bromide according to the protocol of Sambrook and Russell (2001).

### SNP identification and phylogenetic analysis

Single nucleotide polymorphisms (SNPs) were identified using REALPHY (F Bertels, P. B. Rainey, O. K. Silander and E. van Nimwegen, unpublished) with reference to both *Pto* DC3000 and *Psa* V-13 for phylogenetic analyses between all *Psa* strains and within the *Psa* V clade, respectively. A minimum read coverage of 10, a minimum PHRED score of 20 and a proportion of unequal nucleotide sites of less than 5% was used. Alignments of SNPs and all conserved invariant sites were built using bowtie2 (http://bowtie-bio.sourceforge.net/bowtie2/index.shtml) allowing one nucleotide mismatch in each seed alignment (bowtie2 parameter -N). Previously assembled contigs were employed for genomes deposited in Genbank by other groups. Phylogenetic relationships were inferred using RAxML with the GTRGAMMA model (general time reversible substitution model, gamma distributed rate variation) and SplitsTree with 100 bootstrap replicates [39]. Evidence of recombination was obtained initially from SNP alignments against the NZ V-13 genome, followed by Split Decomposition analysis and the Phi test as implemented in SplitsTree. Subsequent analysis used statistical approaches described by Sawyer (1989) [40], and implemented in GENECONV (http://www.math.wustl.edu/~sawyer/geneconv/).

### Calculation of the core and flexible genomes

The core genome of the virulent *Psa* genomes was identified using OrthoMCL, which uses a likelihood algorithm to place genes in ortholog groups [41]. The core genome was defined as those genes present amongst all isolates within the set: species-specific gene family expansion was excluded so that downstream analysis would not be affected by the inclusion of in-paralogs. The size of the clade specific genes refers to any ortholog cluster exclusively present in that clade and no other, however not all members of the clade need have a representative ortholog. Genes private to particular strains or sets of strains were identified using either ALFY [42], or a BLAST approach in which, for a given query and a set of subject genomes, we looked for regions unique to the query by extracting the regions that had no ortholog in the subject genomes. The homology search was carried out using translated BLAST with a maximum E-value of 10^−10^. Only regions of 1 kb or more were considered. This procedure was implemented in the AWK-script “findUniqueBlast.awk”, which is available in Protocol S1.

### Alignment of core orthologs

Alignments were created for each core ortholog group using PRANK (http://code.google.com/p/prank-msa/) alignment software [43], [44]. PRANK improves on classical global alignment methods by applying a phylogeny-aware approach that distinguishes between gaps created by insertions or deletions. Independent insertions are prevented from being matched during the progressive alignment process even when they occur at the same position, and the resulting gaps created by these insertions are not penalized during subsequent alignments [45]. Codon-based alignment was employed, with the guide tree inferred by PRANK from the ortholog sequences and default anchoring of pairwise alignments using Exonerate to speed up the alignment process [46].

### Selection analysis of core genomes

PRANK alignments and trees of core orthologous groups were used as input for selection analyses using Codeml in PAML 4.0. Codeml runs a likelihood calculation for multiple sequences on a phylogeny to estimate the ratio of non-synonymous to synonymous mutations (*ω*) at every codon site. The *ω* ratio is a measure of the direction and magnitude of selection on amino acid changes: *ω*<1 or *ω* = 1 indicate purifying or negative and neutral selection, respectively, and *ω*>1 indicates positive selection [43]. The variation in *ω* among sites is modeled by allowing codons to fall into site classes with *ω* values ranging from 0 to 1 (null model 7) and comparing the results with a more general model allowing codons to have *ω*>1. The likelihood ratio test (LRT) statistic was used to identify the most likely model of sequence evolution at a 1% significance level. Bayes Empirical Bayes estimates of model 8 were then used to identify positive selection acting at individual sites [47], [48]. Subcellular localization was predicted using PSORTb 3.0 for core proteins exhibiting statistically significant signatures of positive selection [49].

### Effector distribution and evolution in *Psa* isolates

The set of type 3 secreted effectors (T3SEs) present in each genome was identified by tBLASTx (E value<1e^−5^, 20% minimum identity) using T3SE sequence queries obtained from the T3SE public database (http://pseudomonas-syringae.org/). The subject database included all virulent and low virulent *Psa* genomes, as well as *P. syringae* pv. *theae* (*Pth*). Partial hits or T3SEs disrupted by contig breaks are recorded as truncated/disrupted effectors. Name assignment of T3SE orthologs was performed using phylogenetic analyses [50]. tBLASTx was subsequently also used to record significant sequence variation between strains including insertion, deletion, frame shifts and/or translocation events.

### Identification of genomic islands in *Psa* NZ V-13

IslandPath and IslandPick, implemented by IslandViewer [51], were used as guides for the identification of genomic islands (GI) in NZ V-13. Six genomes were selected on the basis of parameter cutoffs intended to produce a robust dataset of highly probable GIs: *P. syringae* pv. *syringae* (*Psy*) B728A (NC_007005.1) [52]; *P. fluorescens* F113 (NC_016830.1) [53]; *P. fluorescens* SBW25 (NC_012660.1) [54]; *P. fluorescens* Pf0-1 (NC_007492.2) [54]; *P. protegens* Pf-5 (NC_004129.6) [55]; *P. entomophila* L48 (NC_008027.1) [56]; *P. putida* BIRD-1 (NC_017530.1) [57]. Predicted GIs were subject to manual curation and delineation of the probable GI size using the following criteria: presence of mobile elements such as transposases and integrases; over-representation of virulence-related genes, genes annotated as hypothetical proteins, and/or outbreak clade-specific genes; and the presence of adjacent tRNA genes, which are common phage integration sites [51].

### Growth assay of *Psa* isolates

*A. deliciosa* ‘Hayward’ and *A. chinensis* ‘Hort16A’ plantlets were inoculated with a representative strain from each clade of *Psa* or a stonefruit canker causing isolate of *P. syringae* pv. *morsprunorum* (*Pmp*) to compare bacterial growth and spread in infected tissues. Each strain was cultured overnight on solid KB medium. The next morning, an inoculation solution was prepared by diluting cells in 10 mM MgSO_4_ to a concentration of approximately 10^5^ colony forming units (cfu)/mL. Three-month-old clonally propagated plantlets were inoculated by dipping a needle into the inoculation solution and stabbing the stem, followed by the placement of a 2 µL drop of inoculum on top of the wound site (Figure S1A). Four separate plantlets were stab inoculated for each treatment. Plants were maintained at 17–20°C with a light/dark photoperiod of 14/10 hours. Humidity was constant at 70%. Bacterial growth was monitored subsequent to inoculation on day 0 and on days 4, 8 and 14 in both the site of inoculation and the first leaf above the inoculation site. Stem segments and 1 cm^2^ leaf discs from the base, middle, tip and margin of the leaf (Figure S1B) were surface sterilized in 70% ethanol and individually ground in 200 µL 10 mM MgSO_4_. Serial dilutions of the homogenate were plated on solid KB medium and incubated for two days at 28°C. The cfu calculated was used to determine the cfu/cm^2^ in the stem and leaf tissue.

Statistical analysis involved fitting Analysis of Variance (ANOVA) models using Genstat 14.1 on log_10_ transformed colony count values [58]. This allowed for the model assumption of normally distributed residuals with stable variance to be met. As there were a number of zero observations present in the data, a value of 1 was added to the density data before the log_10_ transformation was applied. For data collected from stems, F-tests were used to assess if the isolate, time, and their interaction effects were statistically significant. Post hoc tests to compare fitted means were also calculated, with Fisher's protected Least Significant Difference (LSD) method used to account for multiple testing. The analyses were carried out separately for each of the two cultivars. The leaf data were analyzed similarly, but also included an area factor that indicated the section of the leaf from which the sample came. The ANOVA tested the area, treatment, and time effects along with all two-way and three-way interactions. In addition, plant ID was treated as a blocking factor because there were multiple measurements on the same plant. All tests were carried out at the 5% level of significance. The back-transformed means are presented in the text with the respective Standard Error Ratio (S.E.R), which is also the back-transformed standard error.

## Results and Discussion

### Complete and draft genome sequencing

High-quality draft sequences were assembled for 23 strains of *Psa* from Illumina data (Table 1). This included five strains from Japan isolated between 1984 and 1988, three from Korea (1997–98), one from Italy (2010) and 14 from NZ (all from 2010). The NZ strains were isolated from plants exhibiting either canker disease or foliar symptoms alone and verified as *Psa* based on diagnostic primers [59]. Strains causing canker disease were consistent with *Psa*-V, whereas the low virulent (LV) form produces mild leaf spotting, but no cankers [28]. In subsequent analyses these 23 draft genomes were supplemented by eleven previously sequenced isolates from China, Italy, Chile and Japan (Table 1).

In addition, the genomes of NZ V-13 and the type strain (J-35) were sequenced with Roche 454 resulting in circularization of each chromosome into a single megascaffold. At approximately 6.5 Mb, both chromosomes are larger than other *P. syringae* pathovars that have been fully sequenced (Table S1). Each strain possesses an autonomously replicating plasmid: NZ V-13 has a 70.1 kb plasmid and the Japanese pathotype strain has one of 32 kb. Restriction digests of plasmid preparations resolved using pulse field gel-electrophoresis (PFGE) matched the predicted pattern and confirm the size of the plasmids (Figure S2). These plasmids are half the size of those reported previously [26].

**Figure 1:**
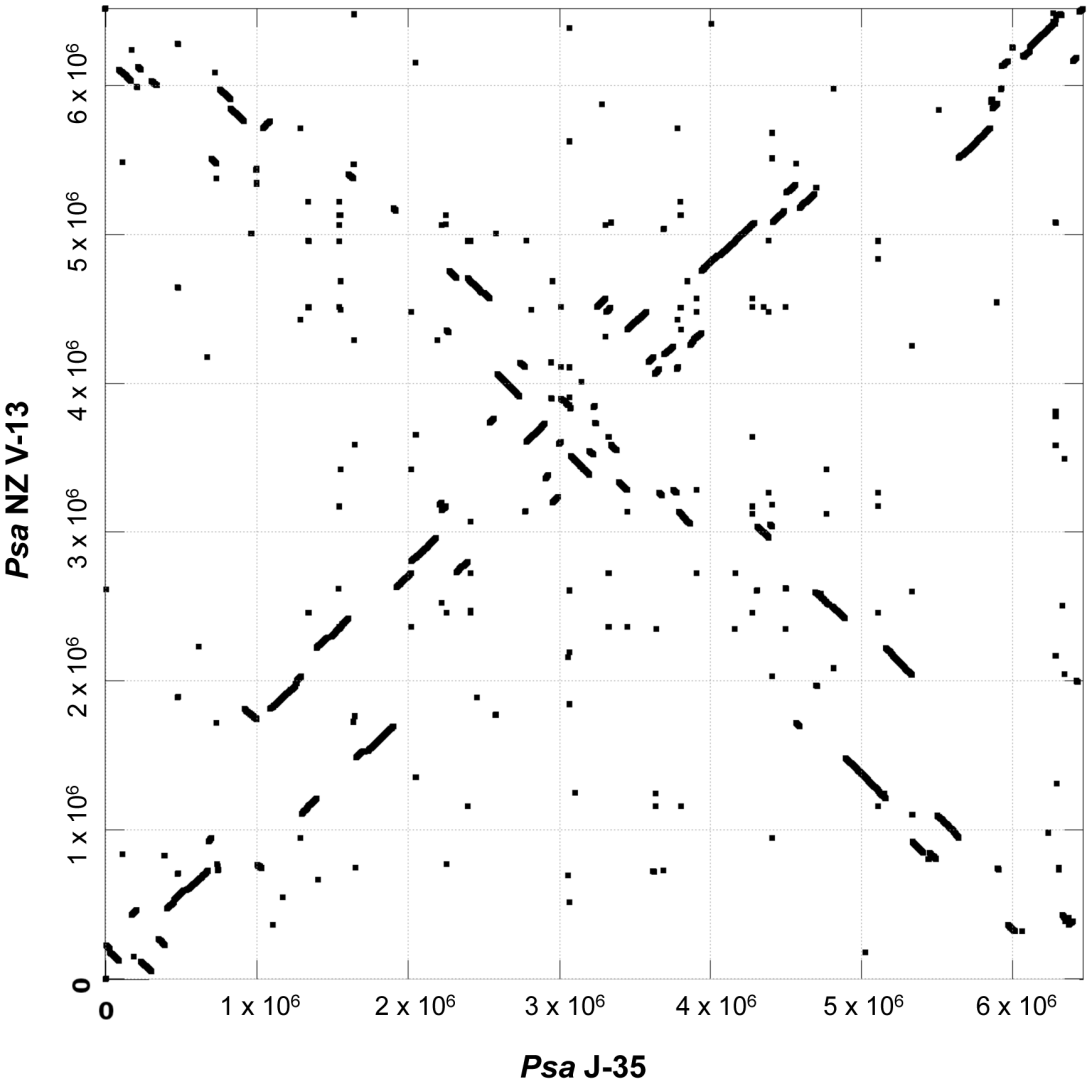
Synteny plot of *Psa* NZ V-13 and *Psa* J-35. MUMmer dotplot displaying stretches of conserved sequence between the genomes of *Psa* NZ V-13 and J-35 as lines with slope = 1. Inverted and translocated stretches of conserved sequence are displayed as lines with slope = −1.

A synteny plot of the two closed genomes of NZ V-13 and J-35 shows evidence of substantial genomic rearrangement (Figure 1). This is unexpected for two strains from a single pathovar. Of particular note is the “X” like structure which indicates multiple translocations and inversions centered about the origin.

### Phylogeny and biogeography

**Figure 2:**
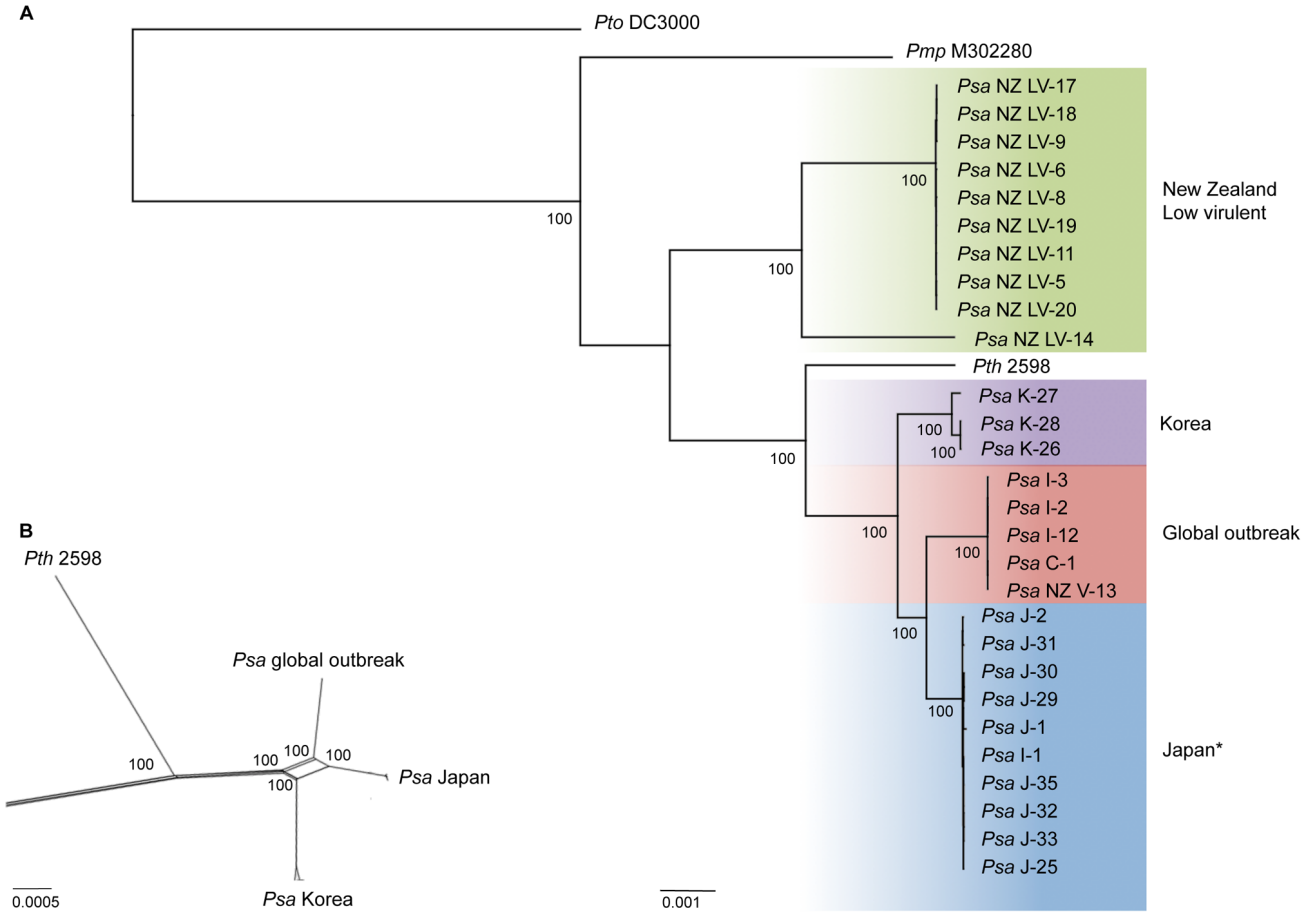
Phylogeny of *Psa* and recombination between canker-causing *Psa* clades. RAxML Maximum likelihood phylogenetic analysis of 32 draft and complete genome sequences based on 15,329 SNPs and 463,396 invariant sites (A). Each phylogenetic group is assigned its own color. With the exception of a single Italian strain (*) isolated in 1992 grouping with the Japanese clade, the canker-causing Japanese, Korean and low-virulent foliar NZ isolates form monophyletic clades reflecting their geographic origin, while global isolates from the 2008–2010 outbreak form a single clade. Bootstrap scores shown are based on 100 replicates. A Splitstree analysis of recombination predicts recombination between canker-causing clades of *Psa* (B). All bootstrap scores are 100 (shown and otherwise).

Initial population analyses were based on single nucleotide polymorphisms (SNPs) obtained by aligning DNA sequence reads to the *Pseudomonas syringae* pv. *tomato* (*Pto*) DC3000 genome (NC_004632.1) [60]. This resulted in 15,329 SNPs and 463,396 invariant sites. Overall diversity as determined by Watterson's theta was 0.008 (per site) [61]. Phylogenetic analyses identified four distinct clades, of which three exhibit strict phylogeographic structure (Figure 2A). The low-virulent (LV) strains responsible for mild symptoms of foliar infection form a single cluster. Leaving aside the divergent NZ LV-14 isolate, the remaining LV strains differ by just 20 SNPs. The three Korean strains, which were isolated in 1997 and 1998, form a clade defined by 147 SNPs. The Japanese strains isolated between 1984 and 1988 also form a monophyletic group (52 SNPs distinguish these strains) and include the *Psa* I-1 strain, which was isolated in 1992 during an early incursion of *Psa* into Italy [17]. The phylogeographic signal displayed by earlier canker-causing and foliar *Psa* is absent from the group of strains isolated from the 2008 outbreak of *Psa* (*Psa*-V): these form a single clade comprising isolates from New Zealand, China and Italy. These strains show little polymorphism, with just five segregating SNPs detected and none separating the *Psa*-V strains from NZ (but see below for results of additional analysis of polymorphism within *Psa*-V).

While each of the four major clades shows little within-clade diversity, the clades are distinguished by substantial SNP diversity. The LV clades differ from the other three clades by ∼4,000 SNPs: the Japanese, Korean and outbreak clades each differ by ∼1,000 SNPs. This level of between-lineage diversity is surprising. While reminiscent of differences among three known *Pto* lineages, contemporary *Pto* is comprised of just a single homogeneous (T1) lineage [62]. Consistent with low diversity, *Pto* T1 shows evidence of repeated selective sweeps with signatures of recurrent arms races with its tomato host [62]. A similar picture exists for *Pph* and *P. syringae* pv. *glycinea* (*Pgy*) [63]. In contrast, different outbreaks of *Psa* have been caused by three related, but nonetheless genetically distinct lineages sampled from a diverse source population. Whether these distinct lineages persist into the future is a matter of interest.

That there are such distinct lineages of *Psa* has significant implications, particularly in light of geographic origin. The first outbreak of canker disease occurred in Japan in 1984 [14], but with the exception of a single transmission event to Italy (recorded in 1992 [17]), the Japanese lineage remained restricted to Japan. Reports of outbreaks in China were also evident in 1984, however no strains are available from these incidents [15]. The second recorded outbreak was from Korea, but our phylogenetic data clearly show that this was not the consequence of transmission from the population established in Japan, but was caused by a separate lineage distinguished by numerous SNPs and hundreds of unique genes (described below). The recent (2008) outbreak of *Psa*-V represents a third independent lineage, again, distinguished from the earlier lineages by large numbers of SNPs and numerous unique genes (described below). Similarly, the LV lineage detected in NZ in 2010 during surveillance for *Psa*-V represents a fourth clade, with the likelihood that *Psa*-LV14 is a fifth (*Psa*-LV14 differs from the other LV strains by ∼1,400 SNPs) [27]. The simplest explanation for this striking genetic structuring is the existence of a discrete but diverse source population from which independent transmission events have led to separate outbreaks of *Psa* disease.

### Within pathovar recombination

While the phylogenetic reconstruction of *Psa* received strong bootstrap support (Figure 2A), we nonetheless explored the possibility of recombination. The Phi test [64], based on 6,346 informative sites, revealed statistically significant evidence of recombination (p<0.0001). Split Decomposition analysis [65] suggests this is a result of recombination among the Japanese, Korean and *Psa*-V strains (Figure 2B) [39]. An analysis based on 5,506 informative SNPs (from these three clades) showed 3,633 are congruent with the phylogeny depicted in Figure 2A, with the remainder being homoplasies due to recombination.

**Figure 3:**
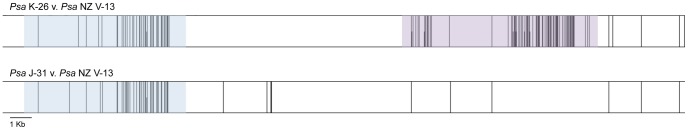
Shared and unique SNPs in *Psa* K-26 and J-31 compared to *Psa* NZ V-13. Sample view from Artemis showing SNPs from *Psa* J-31 and K-26 aligned against a ∼30 kb region of the NZ V-13 genome. Each line represents a SNP that distinguishes J-31 and/or K-26 from NZ V-13. Over this region, K-26 differs from NZ V-13 by 155 SNPs; J-31 differs by 65 SNPs. In the blue region there are 50 SNPs that distinguish J-31 and K-26 from NZ V-13. Of these, J-31 and K-26 are identical at 42 positions. GENECONV predicts that this region represents a gene conversion event between J-31 and K-26. The purple region denotes a gene conversion event into K-26 from a strain outside the set analyzed here. The set of Artemis input files allowing representation of the full set of SNPs, coverage of SNPs and regions identified by GENECONV as statistically supported regions involved in gene conversion events are available as Supplementary Dataset 1.

Homologous recombination among the Japanese, Korean and 2008 (*Psa*-V) outbreak lineages would add further weight to the notion of a source population comprised of a set of co-existing and co-evolving strains. If correct, and if recombination has taken place over sufficiently large genomics tracts, and in recent - as well as past - evolutionary time, then a signature of recombination should be evident by regions of clustered SNPs. To this end we mapped SNPs from a representative Korean (K-26) and Japanese (J-31) strain onto the fully sequenced NZ V-13 genome to clarify the positional distribution of polymorphisms. Visual observation showed highly distinctive patterns of clustered SNPs shared between pairs of strains (Figure 3).

Such striking patterns, reminiscent of admixture in sexual populations, led to a statistical analysis of gene conversion (homologous recombination) events using GENECONV [40], which identifies conversion events between pairs of strains in an alignment (and gene conversion events from strains outside the alignment). The results are shown in Table 2 and confirm the visual observations made above (data files for input into Artemis depicting positions of SNPs arising from comparison between K-26, J-31 and NZ V-13 are available as supplementary data (Dataset S1)). Mapping of the GENECONV-predicted gene conversion events onto the NZV-13 genome showed a close correspondence with regions of highly clustered SNPs (Dataset S1).

**Table 2:** Recombination events between *Psa* strains.

Strain pair^1^	Total length^2^ (kb)	Regions^3^	Average length^4^ (kb)	Proportion of genome^5^	Recombinant SNPs^6^	Proportion of recombinant SNPs
K-26^7^	427.8	172	2.49	0.083	6,085	0.214
J-31^7^	184.4	134	1.38	0.036	2,413	0.085
NZ V-13^7^	302.4	159	1.90	0.059	4,218	0.149
NZ V-13 v. J-31	427.5	173	2.47	0.083	6,073	0.214
K-26 v. J-31	299.8	158	1.90	0.058	4,179	0.147
NZ V-13 v. K-26	183.9	132	1.39	0.036	2,371	0.083

The first three rows depict gene conversion events likely to have arisen from recombination events outside of the.

three compared strains. Rows 4–6 depict gene conversion events likely to have arisen from recombination events.

between pairs of strains (as listed).12,716 unique recombinant SNPs were identified among all strains, out of a total of 28,403 SNPs.

1 Strains used for pairwise comparison of recombination using GENECONV.

2 Total length of genome affected by recombination events.

3 Individual (discrete) regions involved in a recombination event (P<0.05, GENECONV simulation with 10,000 permutations).

4 Average length of recombination event.

5 Proportion of genome affected by recombination.

6 Total identified SNPs.

7 Recombination events predicted by GENECONV to have arisen from outside the analyzed set of three genomes are largely the reciprocal of recombination events identified between pairs of strains.

GENECONV predicts that approximately 10% of each genome is the result of homologous recombination with gene conversion often being between strains included in the data set. For example, Korean and Japanese lineages have exchanged ∼300 kb (158 events of ∼1.9 kb in length) with each other (Table 2). NZ V-13 shares most with J-31 (∼427 kb scattered across 173 regions and comprising ∼2.5 kb per gene conversion event).

While the overall patterns of SNPs, combined with analysis based on Split Decomposition, analysis of homoplasies, the Phi test and GENECONV strongly support the hypothesis that clustered SNPs are a consequence of gene conversion, it is conceivable that these regions might arise through positive or diversifying selection on specific genes, such as those involved in interactions between *Psa* and its plant host [66]. If so, then genes containing highly clustered SNPs are likely to be those with known or suspected roles in arms races with plant resistance genes. A scan of the genome suggests this not to be the case: most clustered regions span housekeeping genes. Nonetheless, using SNAP [67] to estimate numbers of synonymous and non synonymous nucleotide substitutions [68], we tested to see whether regions of the genome identified by GENECONV as resulting from gene conversion had more non-synonymous substitutions compared to regions of the genome identified by GENECONV as being involved in gene conversion. An analysis of the normalized ratio of the proportion of non-synonymous to synonymous substitutions (pN/pS) based on pairwise comparisons of ∼4000 SNPs from K-26, J-31 and NZ V-13 showed pN/pS values less that 1 (the neutral condition) indicative of purifying – and not positive – selection: mean pN/pS (± standard deviation) for putative non-recombinant regions is 0.131±0.004 and for recombinant regions is 0.088±0.002). We therefore conclude that regions of clustered SNPs identified between pairs of strains by GENECONV are a consequence of gene conversion.

Notable from observations of patterns of SNPs mapped onto the NZ V-13 genome (Dataset S1) is the fact that some of the shared regions identified by highly clustered SNPs are identical, or almost identical, between pairs of strains (Figure 3). This indicates that some of the gene conversion events have taken place in recent evolutionary time. This, combined with the fact that each genome carries a signature of gene conversion events scattered around the chromosome, points to the fact that admixture among strains has been a persistent feature of the evolutionary history of these lineages. These patterns of gene conversion events substantiate the notion of a source population. It is difficult to imagine how such patterns could emerge without the sampled lineages having co-existed in close proximity for the bulk of their evolutionary history.

GENECONV provides a measure of the overall contribution of recombination to SNPs, relative to mutation. This ranges from ∼8 to 21% for recombination events due to either gene conversion between known or unknown pairs of strains. Overall, ∼44% of all polymorphic sites are likely to be the result of a recombination event. This means that SNPs are approximately twice as likely to be generated by mutation as by recombination. This level of recombination is twice as high as previously reported for the species [69], but still toward the clonal end of the spectrum of population structures [70].

### SNP analysis of *Psa*-V from the 2008 outbreak lineage

The origin of the current global epidemic is the subject of interest and speculation, with reports that China is the likely source [22], [27], [29]. Previous studies identified six SNPs that distinguished European from Chinese isolates [22]; upon inclusion of two Chilean isolates, nine diagnostic SNPs were identified [27]. Our sequencing of multiple additional isolates of *Psa*-V confirms that the 2008 outbreak is from a single clone that has rapidly spread around the globe [22]. Whereas Mazzaglia et al [22] relied on read-mapping *Psa*-V to the partial genome sequence of *Pth* and Butler et al [27] adopted a similar approach using DC3000 as the reference genome, we were able to map reads to the complete genome of NZ V-13. We also included the draft genome of the divergent Chinese strain C-9 (M228) in our analysis [27].

Overall we identified 979 SNPs – many of which are unique to C-9: 515 SNPs distinguish Italian, Chinese (excluding C-9), Chilean and New Zealand *Psa*-V strains (Table 3). However, most of these SNPs are located within integrative conjugative elements (see below) and are therefore the result of lateral gene transfer (the number of SNPs across these elements is in the tens of thousands, but are too polymorphic to be detected via read-mapping). Because SNPs due to recombination stand to mislead phylogenetic analyses recombinant SNPs were removed (Table 3) for subsequent analyses.

**Table 3:** SNPs distinguishing *Psa*-V isolates.

	*Psa* I-2	*Psa* I-3	*Psa* I-10	*Psa* I-12	*Psa* Cl-4	*Psa* Cl-5	*Psa* V-13	*Psa* C-1	*Psa* C-9^1^
*Psa* I-2	0	52	28	36	37	34	38	38	365
*Psa* I-3	71 (19)	0	32	40	41	38	42	42	369
*Psa* I-10	45 (17)	34 (2)	0	16	17	14	18	18	345
*Psa* I-12	60 (24)	49 (9)	23 (7)	0	25	22	26	26	353
*Psa* Cl-4	115 (78)	104 (63)	78 (61)	93 (68)	0	5	23	23	350
*Psa* Cl-5	112 (78)	101 (63)	75 (61)	90 (68)	5 (0)	0	20	20	347
*Psa* V-13	346 (308)	335 (293)	309 (291)	324 (298)	337 (314)	334 (314)	0	24	351
*Psa* C-1	345 (307)	334 (296)	308 (290)	323 (297)	336 (313)	333 (313)	25 (1)	0	351
*Psa* C-9	558 (193)	547 (178)	521 (176)	536 (183)	587 (237)	584 (237)	818 (467)	817 (466)	0

SNPs due to mutation appear above the diagonal; total number of SNPs appear below the diagonal with the number due to recombination included in brackets.

**Figure 4:**
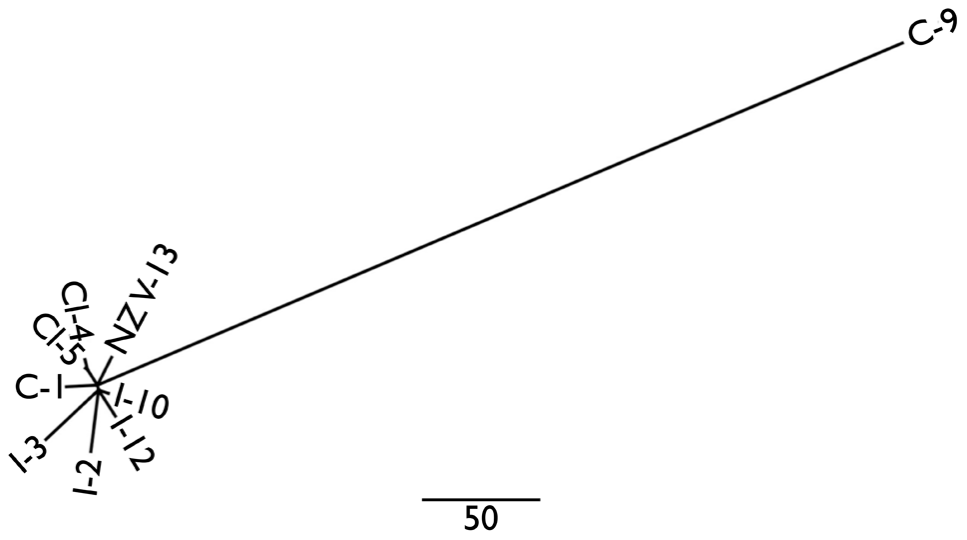
Phylogeny of *Psa*-V isolates and the divergent Chinese isolate C-9. Neighbor joining tree of *Psa*-V and C-9 isolates built in PHYLIP using SNPs due to mutation alone. These distances are also displayed in the upper right section of Table 3.

1 *Psa* C-9 is included here for comparative purposes but is divergent from the global outbreak strains, as shown in Figure 4.

As evident in Figure 4, the Chinese strain C-9 is highly divergent [27]. It shares no SNPs with any of the other *Psa*-V isolates. On the basis of the available data – and excluding SNPs due to recombination – C-9 differs from C-1 by 351 SNPs (and other *Psa*-V strains by similar numbers).

Leaving aside the Chinese strain C-9, the Italian strains encompass the greatest level of polymorphism – a finding confirmed through SNP analysis of the genomes of six newly acquired (unpublished) Italian strains. This is consistent with the fact that *Psa*-V was first reported in Italy [17]. The Chilean strains are distinguished by five SNPs. No SNPs were identified among the five NZ *Psa*-V strains isolated immediately after the outbreak in November 2010. Of the SNPs that distinguish the four Italian strains, two are shared. No SNPs are shared between NZ V-13 and C-1. The lack of informative sites means the phylogeny of *Psa*-V is star-like (Figure 4). The contemporary isolation of the divergent C-9 isolate is consistent with the suggestion that *Psa* is endemic on wild *Actinidia* relatives [27]. However, while C-9 clearly shares a common ancestor with *Psa*-V, C-9 is not the source of the global outbreak.

Assuming a mutation rate in the order of ∼10^−7^ SNPs per site per year [32], [71] and the fact that the most divergent clones differ by 52 SNPs, establishment of the *Psa*-V clone is likely to have occurred less than 10 years ago. C-9 is likely to have diverged from the outbreak clade ∼100 years ago.

### Identification of the core and accessory genomes

**Figure 5:**
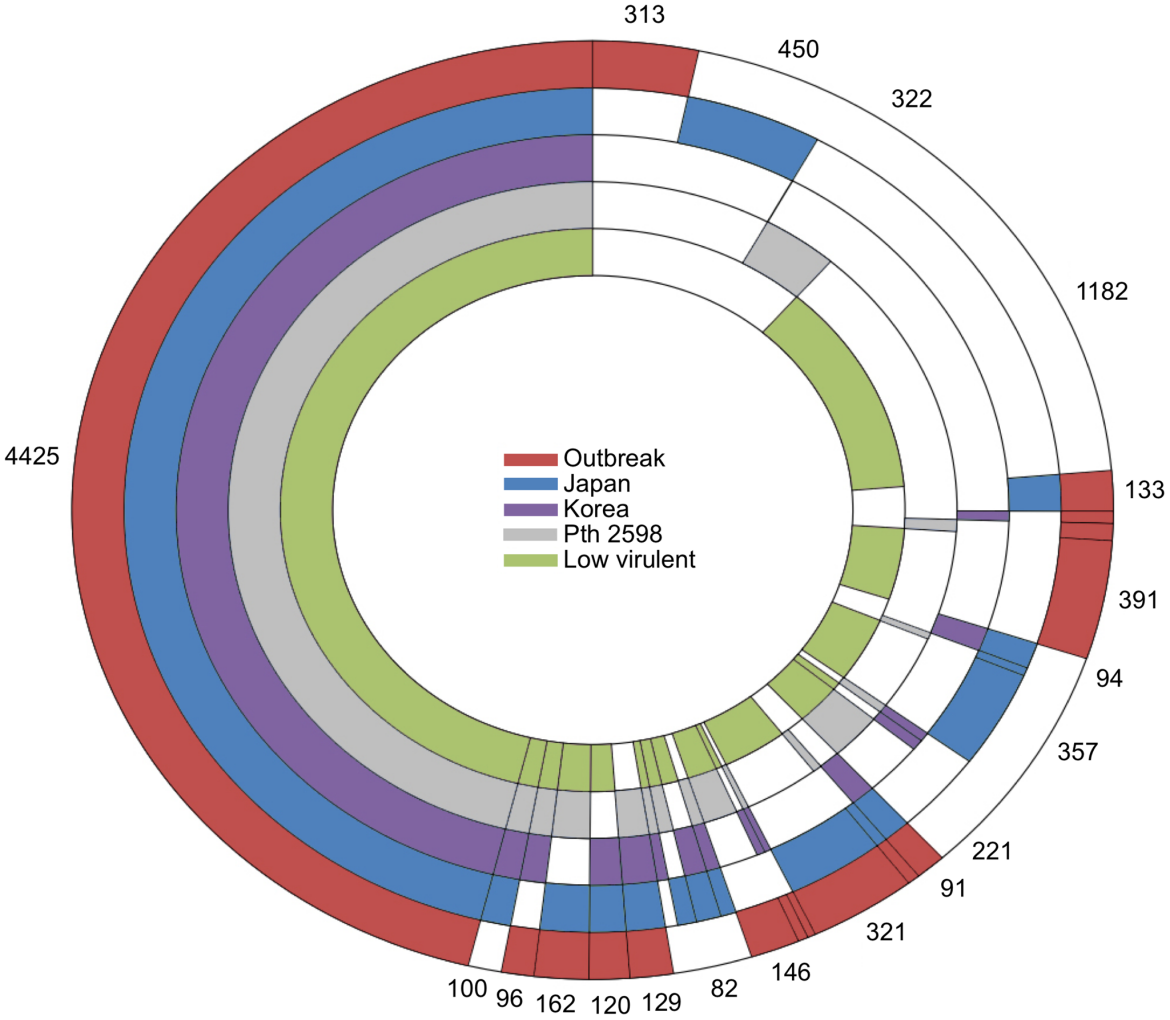
Unique and shared ortholog groups between clades. Numbers outside the rainbow plot show the number of ortholog groups with at least one representative ORFs per strain per clade.

Identification of genes in the accessory pool often provides clues as to traits of ecological significance. Although there is considerable gene conservation within each *Psa* clade, there is also evidence of extensive gain and loss. The core genome of the *Psa*-LV, *Pth* 2598 and canker-causing Korean, Japanese and outbreak *Psa*-V strains includes 4425 orthologs, and the flexible genome includes an additional 4710 orthologs (Figure 5). The *P. syringae* core genome encodes many proteins contributing to its success in the phyllosphere and endophytic environments, however it is likely that some of these core proteins may present microbe-associated molecular patterns (MAMPs) recognized by plant host receptors [72]. MAMP-triggered immunity limits bacterial growth *in planta*, and can result in selection for amino acid diversification in elicitor-active regions of conserved pathogen proteins [73].

Of the 313 orthologs represented once or more among *Psa*-V strains, a set of 137 orthologs are found in all outbreak strains, and are absent from the Korean and Japanese *Psa* clades (Figure 5). These genes do not share a common history. The most similar orthologs of 39% of these genes are found in different pathovars across 4 different phylogroups of *P. syringae* and the remainder have close orthologs in plant-growth promoting soil bacteria as well as aquatic and insect associated bacteria, reflecting *P. syringae*'s cosmopolitan lifestyle (Table S3) [72]. Almost 10% of the outbreak-specific genes are annotated as phage integrases or transposases.

There are 18 genes present solely within outbreak strains which have top BLAST matches to known vascular and woody pathogens: these represent candidates most likely to confer a virulence advantage to *Psa*-V. These include genes from *P. syringae* pv. *lachrymans* (*Pla*) and *P. syringae* pv. *pisi* (*Ppi*), which invade vascular tissues, and the strains responsible for bacterial canker disease in horse chestnut, plum, and citrus trees (*P. syringae* pv. *aesculi* (*Pae*); *Pmp* and *Xanthomonas axonopodis* pv. *citri* str. 306, respectively) [74]–[77].

### Diversifying selection in the core genome

An analysis of selection based on the core genome of all three canker-causing clades of *Psa* revealed 17 genes that exhibit significant signatures of positive selection (Table S2). Five of these candidates have non-synonymous substitutions exclusive to the outbreak clade, representing a shortlist of genes whose signatures of diversifying selection could be due to interactions with pattern-recognition receptors of *Actinidia* species. Curiously, this shortlist includes the flagellar P-ring protein FlgI, which forms a ring structure of the basal body in the peptidoglycan layer. The flagellar filament, comprised of multiple subunits of FliC, is known to harbor an epitope that induces a strong innate immune response in plants [62], but there are no reports suggesting this of FlgI [78]. Seven of the 17 candidates with high-confidence predictions of subcellular localization are targeted to different layers of the bacterial cell wall, consistent with their potential function as MAMPs [49].

### The plasmid from *Psa* NZ V-13 possesses novel effector fusions and genes found in pathogens of woody plants

The NZ V-13 70.1 kb plasmid is a low copy number plasmid based on the small amount of DNA extracted from large-scale plasmid preparations. Consistent with this is the presence of the *stbD* and *stbE* genes which encode a putative segregational stability mechanism probably encoding a toxin-antitoxin system [79]. Analysis of the genes in the plasmid revealed a number of novel features. It possesses three genes encoding predicted T3SEs, HopAU1, HopAV1 and HopAA1-2. Two of these effectors possess novel fusions or insertions. HopAV1 has a C-terminal in-frame fusion to a TrbC conjugal transfer protein; and HopAA1-2 has a 106 bp insertion causing a frame-shift. The plasmid also possesses two adjacent gene clusters involved in aromatic carbon metabolism. Both pathways are found in the woody pathogen *Pae* [72], and some of the genes are present in the xylem-limited pathogen *Xylella fastidiosa* [80]. The first cluster consists of two genes involved in the biosynthesis of anthranilate. The second cluster has the hallmarks of a secondary metabolic pathway for a secreted compound including putative regulatory and efflux proteins plus three genes with predicted roles in secondary metabolism. Intriguingly, the first of these is a putative phenylacetate CoA ligase for which anthranilate may be a substrate. It suggests this cluster is making an active compound with a role in the infection of vascular tissue.

### Genomic and pathogenicity islands in *Psa*-V

Many outbreak-specific genes are found clustered with mobile genetic elements in genomic and pathogenicity islands (GIs, PAIs), which are frequently linked to virulence and other environmental adaptations [51], [81]. Sixteen genomic and pathogenicity islands (PAI) ranging in length from 10 to 100 kb were identified in NZ V-13 with IslandViewer. The PAIs are enriched with T3SEs as well as outbreak-specific genes. In addition, there are two prophages with homology to PSPPH06 and a Mu-like prophage. A total of 15 transposons harboring T3SEs or outbreak-specific genes were also identified.

### Integrative and conjugative elements

Mazzaglia et al [22] identified a polymorphic region with similarity to PPHGI-1, an integrative and conjugative element (ICE) identified in *Pph* 1302A [82]. This element was further characterized and shown to be capable of excision from the genome and circularization [27]. A hagfish plot mapping the Illumina reads from this region in *Psa* NZ V-13 showed no significant additional coverage compared to the rest of the genome suggesting that it is present as a single copy per genome (Figure S3).

**Figure 6:**
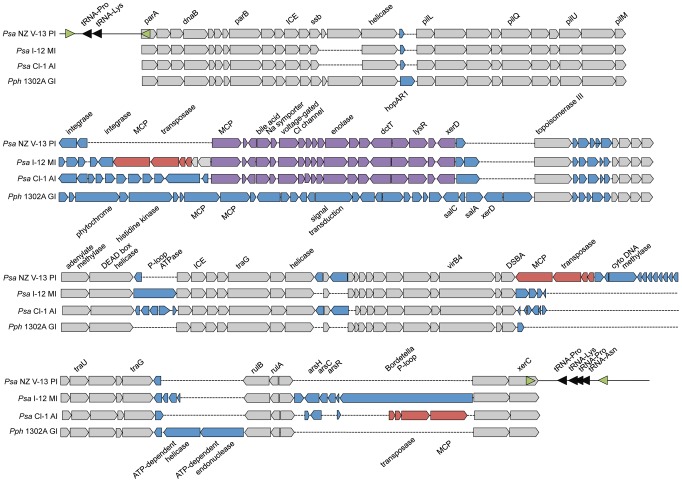
Structure of the Pacific, Mediterranean, Andean and PPHGI-1 islands. Grey genes are orthologs with ∼75% nucleotide identity. Blue genes are variable accessory genes. Purple genes are accessory genes with complete conservation across the Pacific, Andean and Mediterranean islands. Red genes have translocated *via* transposon-mediated insertion events. Each island is bounded by 52 bp *att* sequences overlapping tRNA-Lys. Primer sites for the confirmation of excision and chromosomal integration are shown in green (Figure S4).

Detailed analyses of the island present in *Psa* NZ V-13, referred to as the Pacific Island, revealed a 100 kb region comprised of ∼100 predicted open reading frames bounded by *parA* (left) and *xerC* (right) and integrated at a 52 base pair *att* locus (*att*-1) within a tRNA^(lys)^ (Figure 6). Another copy of this *att* site (*att*-2) was found in the *Psa* NZ V-13 genome 2.7Mb from *att*-1. This site appears to be unoccupied in *Psa* NZ V-13, however the ICE from *Psa* I-12 is located at *att*-2 and the corresponding *att*-1 site is empty (Figure S4).

Comparison with the PPHGI-1 ICE shows striking similarities and marked differences. The “core” genes from the *Psa* NZ V-13 ICE are syntenous with PPHGI-1, although they share only ∼75% nucleotide identity. Saturation at the third codon position indicates these elements have an ancient evolutionary past. PPHGI-1 carries a number of accessory genes that are not present in the Pacific Island, which for example lacks the *hopAR1* effector. The Pacific Island also carries a novel set of genes located between the Pil locus and the conserved topoisomerase III (purple genes in Figure 6). This set of genes includes a predicted enolase and various transporters, including an ortholog of *dctT*, a putative di-carboxylic acid transporter and a methyl-accepting chemotaxis protein (MCP) predicted to be involved in taxis toward malate. Interestingly, the DctT transporter carries, at its N-terminus, a sequence predicted by SecretomeP version 2.0 to be used to target proteins for secretion (SecP score = 0.64 [83]). Analysis using EffectiveT3 to predict proteins targeted to the type 3 secretion system (T3SS) (trained on effector sequences from DC3000) returned a highly significant probability score (0.97), strongly suggestive of type 3-targeting [84]. In accordance with this prediction we note that 14% of the first 50 amino acids of the protein are either Ser or Pro [85]. No other transporter in this region of accessory genes carries a similar signal.

While this set of accessory genes awaits experimental analysis, putative functions of a number of the predicted proteins together suggest a role in manipulation of host cell metabolism. Pseudomonas has a preference for growth on TCA cycle intermediates and *dctA1* a dicarboxylate transporter gene is important for the virulence of *Pto* DC3000 [86]. It is possible that the putative T3SS-targeted di-carboxylic acid transporter, enters the plant cell and incorporates into a host cell membranes to facilitate export of this sugar acid in a manner analogous to that recently suggested for á keto glutarate in *Xanthomonas oryzae* pv. *oryzae* [87]. Enolase is the penultimate step in glycolysis and the enzyme is exported as part of the RNA degradosome [88]. The role of the enolase in the ICE is not clear. One possibility is that it plays a role in the conversion of dicarboxylic acids to glucose, alternatively it might enhance activity of glycolysis in plant cells. In this regard it is of interest to note that a study of proteins differentially expressed in olive, with and without the pathogen *P. savastanoi* included *dctT* and plant enolase [89].

The NZ V-13 ICE is also present in the genome of the Chinese outbreak strain C-1, however, as previously indicated [22], [29], this region is highly divergent in the four Italian and two Chilean outbreak strains [27]. A comparison of the Italian (Mediterranean Island), Chilean (Andean Island) and the Pacific Island is depicted in Figure 6.

Several notable findings emerge from the comparison of these Islands. Firstly, the core genes of the *Psa* islands are syntenous with those of PPGHI-1, but divergent at the nucleotide level. In fact the core genes of the Mediterranean Island are no more similar to the Pacific Island core genes as to PPHGI-1. Secondly, the enolase/*dctT* region is present all three *Psa* Islands and differs by just four SNPs. This suggests that this region has a recent evolutionary history and has an important function in *Psa* virulence. Thirdly, while there is near identity at the enolase/*dctT* locus there are a number of significant differences in the accessory genes. Most notable is the presence of a putative P-loop protein encoded by a gene of ∼6 kb with highest similarity to a gene found in *Bordetella* (Figure 6). Finally, all *Psa* Islands carry an identical MCP/transposase region (red genes in Figure 6), which has recently been translocated – presumably via the associated transposon – in one or other of the ICEs. The picture of evolutionary change encompassed by these three laterally transferred ICEs is remarkable. Each displays a distinctly different evolutionary history that is independent of the host genome. Nonetheless, the presence of the conserved enolase/*dctT* region suggests that accessory gene content maybe determined by host-driven selection. Use of such dynamic elements as the basis upon which to infer *Psa*-V phylogeny is likely to generate erroneous conclusions.

### Extensive variation of *Psa* effector repertoires

**Figure 7:**
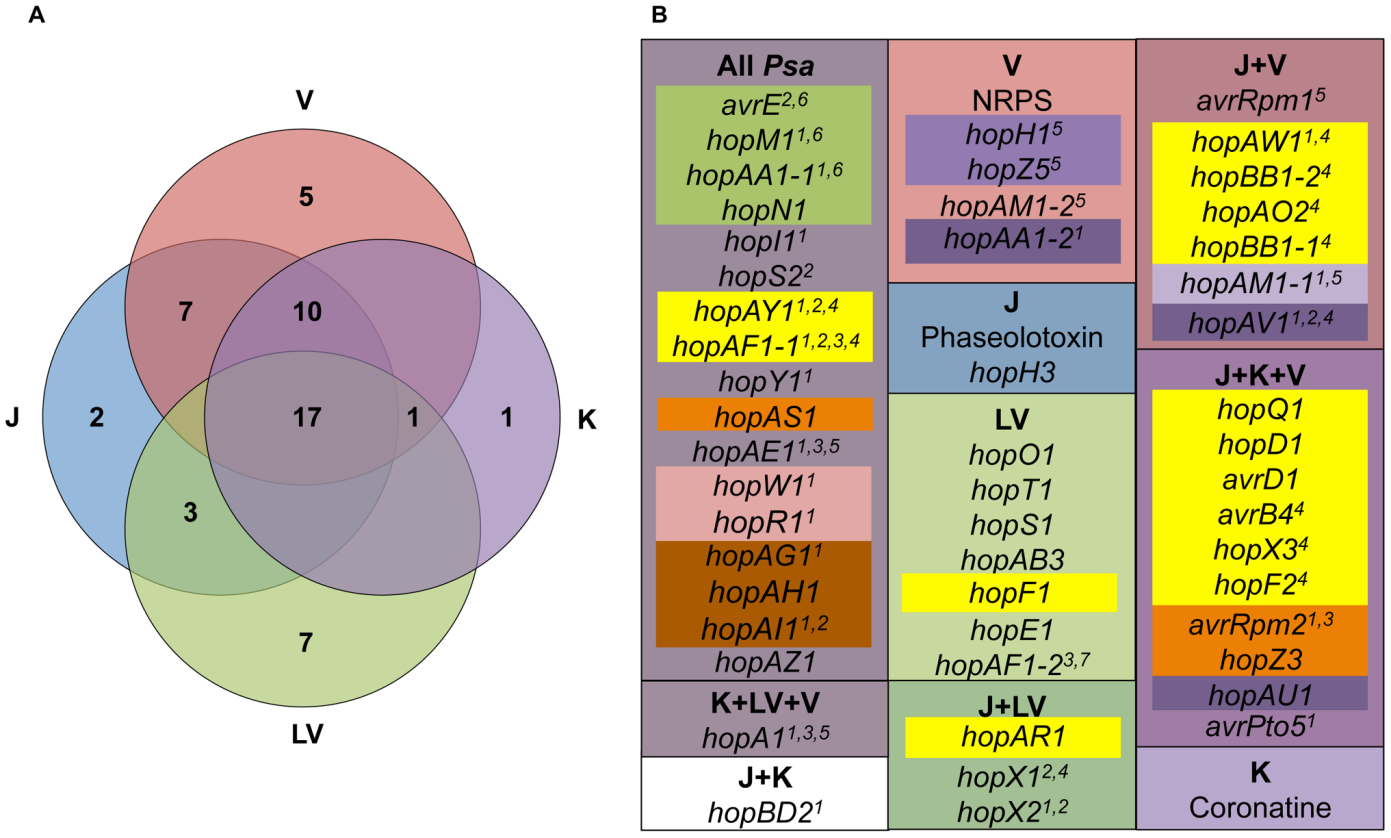
Type 3 secreted effector and toxin distribution in *Psa* clades. The numbers inside each region of the Venn diagram represent T3SEs with orthologs present in the low-virulent (LV), Korean (K), Japanese (J) and recent global outbreak (V) clades (A). The outer boxes in B reflect the clade-specific distribution of T3SEs (italicized) and toxins, while the color of the internal text boxes refers to their occurrence on genomic islands. ^1^premature terminations, partial translocations, frame shifts, and out of frame indels in some strains. ^2^variation between alleles (>2% variation, in frame fusions/indels). ^3^indicates the T3SE may not be in all *Psa* K strains. ^4^indicates the T3SE may not be in all *Psa* J strains. ^5^T3SE that occur within a transposon region. ^6^T3SE is present in the conserved effector locus. ^7^LV has two effectors in the HopAF1 group, HopAF1-2 is most closely related to HopAF1-1 in the Korean clade while HopAF1-1 is most closely related to HopAF1-1 in the outbreak and Japanese clades.

The sequenced *Psa* strains carry 51 known T3SEs (Figures 7 and S5). Seventeen T3SEs are found in all *Psa* genomes, while strains in the outbreak clade carry an average of 39 effectors, and strains in the low-virulent (LV) clade carry an average of 28. Many *Psa* T3SEs are not predicted to be translocated or functional due to the presence of frameshift mutations and transposon insertions. The effector complement is thus highly dynamic even within a single pathovar. T3SE gain, loss and adaptation are likely to have contributed to the enhanced virulence of the outbreak lineage. A large proportion of the variation in effector complement has occurred in a single pathogenicity island encompassing a locus with features similar to that described by Alfano et al, which we have therefore referred to as the exchangeable effector locus (EEL) [90]. Table S4 presents a comparison between effectors at the EEL across several isolates and suggests multiple re-arrangements, insertions and deletions have occurred in and around this island. The outbreak EEL is the largest with 16 T3SEs and the non-ribosomal peptide synthase (NRPS) cluster (Table S4). The pathotype strain *Psa* J-35 EEL is missing the NRPS cluster and has 13 T3SEs, while the Korean and low-virulent clades have fewer T3SEs in this region. The EEL appears to be absent from *Pth* altogether, although *hopAY1* is present. Three effectors in the Japanese and outbreak EEL (*hopBB1-1*, *hopBB1-2*, *hopX3*) share both a chaperone (*shcF*) and their predicted proteins share significant N-terminal sequence similarity to HopF2, as has been observed for AvrRpm2.

**Figure 8:**
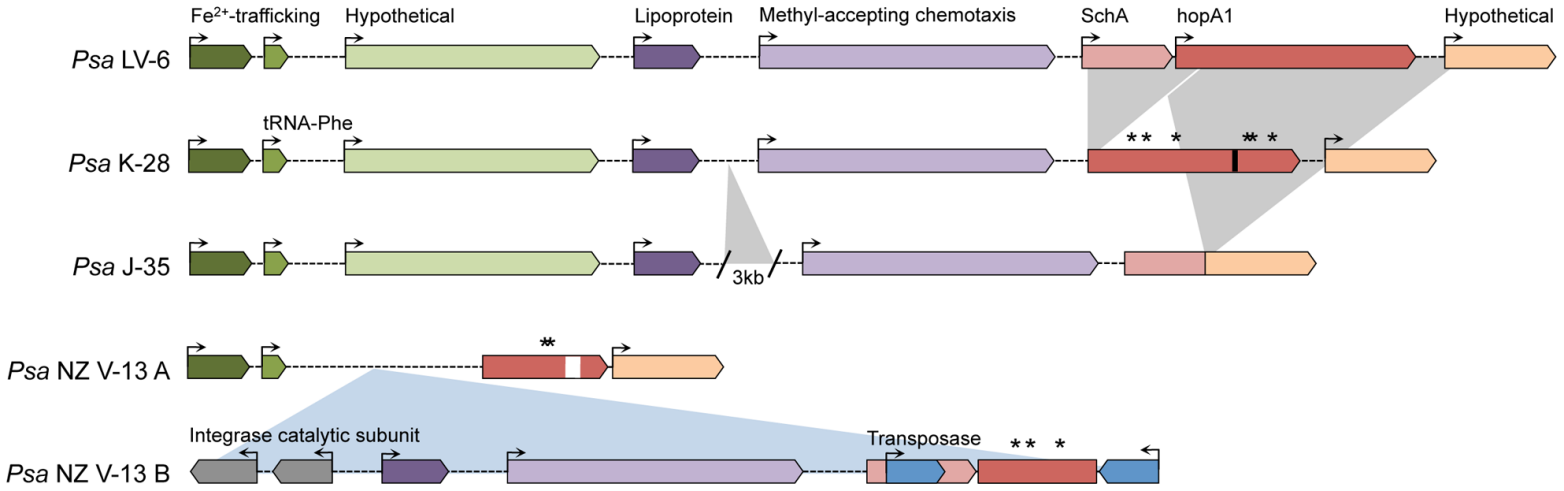
Rearrangements, insertions and deletions in the *hopA1* locus. The deletion of the *schA* chaperone and N-terminus of *hopA1* in K-28 and deletion of *hopA1* in J-35 are displayed with grey triangles. The transposon-mediated excision of a region in the *hopA1* locus and its reintegration 500 kb away in the *Psa* NZ V-13 genome is shown with a blue triangle. Arrows at the 5′ end of the coding sequence indicate which alleles are functional. Stars above *hopA1* indicate the presence of non-synonymous (NS) mutations. The white bar in NZ V-13 refers to a deletion and the black bar in *Psa* K-28 indicates the position of a nonsense mutation in *hopA1*. The deletion in NZ V-13 includes one of the non-synonymous mutations in K-28.

The disruption and loss of effectors may play a role in modulating virulence of the outbreak clade since this can result in loss of host recognition. HopA1, for example, has been inactivated in multiple strains of *Psa* (Figure 8). A deletion of *shcA* and the 5′ fragment of *hopA1* has occurred in *Psa* K-28; the fusion of *shcA* with an unknown gene downstream of *hopA1* has resulted in the deletion of *hopA1* in *Psa* J-35; and lastly, a transposon-mediated translocation has moved the 5′ region of *hopA1* 600 kb away in *Psa* NZ V-13. In *Psa* K-26 *hopA1* contains six non-synonymous mutations; five of these are also present in the fragments of *hopA1* found in *Psa* NZ V-13. Six codons were also deleted in this strain; surprisingly, in the same position as the sixth substitution in *Psa* K-28. Only the NZ LV clade appears to have a fully functional version of HopA1, suggesting the inactivation of this effector contributes to vascular infection, potentially by abolishing resistance gene-mediated recognition. HopA1 is known to disrupt an *Arabidopsis* protein complex formed by EDS1, a central regulator of immunity, and RPS6, a NB-LRR resistance gene, activating effector-triggered immunity (ETI) [91]. Interestingly, HopA1-triggered immunity has also been shown to block the HopM1-mediated degradation of an *Arabidopsis* protein (AtMIN7) linked with vesicle transport and anti-microbial compound secretion [92].

HopM1 is broadly distributed among *P. syringae* pathovars and makes an important contribution to pathogen virulence by degrading AtMIN7. Inactivation of this key host target severely compromises both PAMP and effector triggered immunity (PTI, ETI) [92]. The transposon-mediated disruption of HopA1 in the outbreak strain may thus abolish ETI and allow HopM1 to interact with an *Actinidia* ortholog of AtMIN7. Transposon movement is doubly implicated in the enhanced virulence and transmissibility of the outbreak strain. The transposon-mediated introduction of HopH1 may have suppressed HopA1-induced ETI, as it has been shown to do in *Nicotiana* [93].

A small set of effectors appears to have been gained by the outbreak clade (and are largely absent from the J, K, and LV clades). This includes *hopH1* and *hopZ5*: both effectors are adjacent to each other on a small transposon, suggesting introduction by a lateral transfer event. *hopZ5* is a new member of the YopJ acetyltransferase family and shares greatest similarity to homologs from *Acidovorax* and *Xanthomonas*, although both these lack the putative myristoylation site (Figure S6). Members of the YopJ acetyltransferase family are believed to impede protein kinases by trans-acetylating key serine or threonine residues in the kinase activation loop [94]. Recently HopZ1a has also been shown to interfere with microtubule assembly in the host, perhaps by binding to and acetylating tubulin – or indirectly via its effect on kinases as detailed above [95]. Another HopZ1 family member has been shown to interact with a protein involved in isoflavone biosynthesis, resulting in increased host susceptibility [96].

### Differences in virulence between *Psa* strains

The identification of the effector repertoires and outbreak-specific genes may be linked with differences in host-specificity demonstrated by the growth of *Psa* strains on different kiwifruit cultivars. Growth assays of representative isolates from the low-virulent clade (NZ LV-5) and canker-causing Korean (K-26), Japanese (J-35), and outbreak (NZ V-13) were performed in *A. deliciosa* cv. ‘Hayward’ and *A. chinensis* ‘Hort16A’ in order to understand dynamics of *in planta* growth. Assays involved regular quantification of bacterial density at both the site of initial inoculation (stem) and at three spatially distinct regions of the first leaf above the inoculation point (Figure S1B). This latter measure provides a quantitative indication of the capacity of a given strain to spread *in planta*. *Pmp*, which causes canker in *Prunus* spp., was included as a negative control.

**Figure 9:**
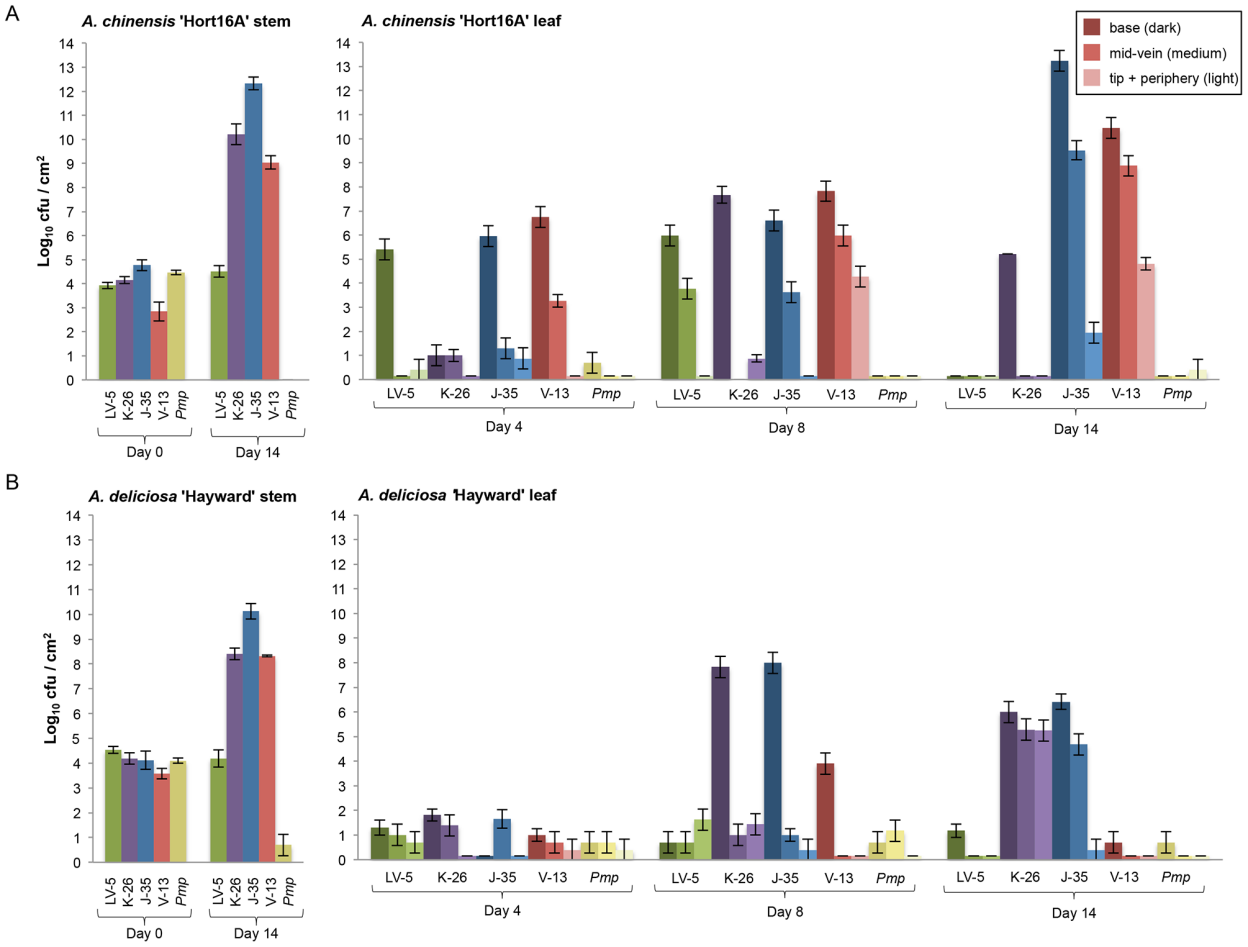
Pathogenicity assay of *Psa and Pmp* strains on kiwifruit. The growth of the canker-causing *Psa* J-35 (blue), NZ V-13 (red), and K-26 (purple) isolates was assayed on the ‘Hort16A’ (A) and ‘Hayward’ (B) cultivars of kiwifruit, along with the low-virulent NZ LV-5 (green) and a strain of *P. syringae* pv. *morsprunorum* (*Pmp*, yellow),that causes canker disease in *Prunus* spp. The average bacterial density (cfu ± SE) was assayed in the stem tissue at day 0 immediately following stab inoculation, as well as in the base of the first leaf above the inoculation site (no *Psa* or *Pmp* observed, data not shown). The bacterial density was quantified in the base of the first leaf above the inoculation site (dark colored bar), the center of the leaf along the mid-vein (medium colored bar), and at the leaf tip and periphery (light colored bar) 4, 8 and 14 days after inoculation. A mock inoculation with MgSO_4_ buffer was also performed, no *Psa* or *Pmp* growth was observed (not shown).

Differences among treatments were tested using a three-way ANOVA within each cultivar, testing for effects of isolate (NZ V-13, NZ LV-5, J-35, K-26 and *Pmp*), time (days 0, 4, 8 and 14) and sample location (stem (point of initial inoculation), base of first leaf, middle of first leaf and periphery of first leaf), with Fisher's protected LSD post-hoc test at α = 0.05 (Supplementary Table S5). On ‘Hort16A’ J-35 reached highest cell densities in the leaf at day 14, however, NZ V-13 spread more rapidly reaching the leaf tip by day eight (Figure 9A). K-26 showed reduced capacity for both growth and *in planta* spread. This capacity was further reduced in NZ LV-5. *Pmp* failed to spread from the point of inoculation, or persist within stem tissue, providing a compelling example of the effectiveness of non-host resistance in vascular plant tissue.

On cultivar ‘Hayward’ all strains showed a reduction in both growth and *in planta* spread (relative to ‘Hort16A’). This was particularly striking for NZ V-13 which attained moderate leaf population densities by day eight, failed to spread beyond the leaf base, and was barely detectable by day 14 (Figure 9B).

Overall these data provide evidence of cultivar-specific differences in the growth dynamics of *Psa* from different clades. Noticeable is the enhanced growth and spread of NZ V-13 on Hort16A relative to its performance on ‘Hayward’. This apparent tradeoff is suggestive of adaptation of this *Psa* lineage to *A. chinensis*. J-35, however, was equally capable of colonizing both ‘Hort16A’ and ‘Hayward’, although showed a reduced capacity for *in planta* spread. This suggests that differences in the gene content between NZ V-13 and J-35 may be central to understanding the enhanced transmission of 2008 outbreak strains both within and between plants [28].

While differences in the growth of all three canker-causing lineages were evident, similarities were also apparent. For example, all three, despite substantial difference in accessory gene context, were capable of spread and persistence within both kiwifruit cultivars. In light of the predicted existence of an Asian source population this finding has special relevance. The fact that three strains with such different genomes can each grow *in planta* raises the possibility that the capacity to cause disease in kiwifruit resides primarily in the core – as opposed to accessory – genome. If so, then it is possible that numerous different strains from the source population may be able to cause disease in kiwifruit. This highlights the importance of understanding the source population and minimizing the chance of future transmission events from this reservoir. It also suggests that strategies to develop durable resistance should not focus solely on effectors or other typically targeted components of the accessory genome.

### Conclusions

The origins of crop diseases are linked to domestication of plants [3]. Since most crops were brought under domestication many centuries ago, opportunities to understand the emergence of disease are limited. Kiwifruit is an exception: it is one of the few plants to have been domesticated in the 20^th^ Century [12].

Our in-depth genomic and population analysis of more than 30 *Psa* strains from diseased kiwifruit vines – strains representing each of three major disease outbreaks obtained from different geographic regions over the course of three decades – has captured initial stages in the emergence of a pathogen population concomitant with domestication of its host. Particularly striking is the genetic structure of the *Psa* population. Rather than a single genetically homogeneous lineage that establishes in one region before transmission to another, each outbreak of *Psa* represents a distinct lineage with its own unique repertoire of accessory genes including effectors and toxins. There can be little doubt that each lineage represents an independent sampling from a single source population. This is evident in the shared ancestry (they form a single monophyletic group), the marked signatures of within pathovar gene conversion, and lack of diversity within each disease-causing lineage.

The geographic location of the predicted source population and the extent of diversity encompassed awaits elucidation, nonetheless, given the recent timing of kiwifruit domestication it is likely that the source population is associated with wild *Actinidia* species in Asia – a finding supported by the divergence of strain C-9 [27]. The diversity of genotype and virulence is likely substantive given knowledge of the heterogeneous characteristics of the lineages studied thus far. For example, the LV isolates from New Zealand cause leaf spot but minimal damage to vines, whereas *Psa*-V isolates cause cankers and stem wilt, are highly transmissible, and have devastating effects on vine health. Interestingly, the source population encompasses *Pth*, the tea pathogen. Given that both hosts, tea and kiwifruit, have their origins in Asia, this finding is perhaps not surprising. Whether existence of two different pathovars within a single lineage represents an instance of host-shift or divergence from within the source population is unclear, but nonetheless highlights the importance of defining the nature of the source population.

The concept of a source population from which disease arises by sampling events has implications for both disease control and plant breeding. From the perspective of disease control there are two interrelated issues. The first is opportunity for transmission from the source population, the second is opportunity for a given transmission event to result in establishment of a new population. Every effort needs to be made to ensure that routes of transmission from the source population to commercial orchards – and between commercial orchards – are minimized. This includes protocols to prevent transmission of Japanese and Korean lineages, which our growth data show could be equally problematic, were they to arrive in countries such as New Zealand from which these lineages are currently absent. At the same time, there is need to move from orchards comprised of single clone varieties in order to reduce opportunity for plant-to-plant transmission and the establishment of high pathogen titers. It is possible that densely planted orchards of *A. chinensis* in Italy were central to the establishment and global dissemination of *Psa*-V.

Challenges to plant breeding are potentially more significant. Producing kiwifruit vines resistant to *Psa*-V is an important goal, however, resistance is unlikely to be durable if breeding programs exclusively target this lineage. The extensive diversity of effectors and associated virulence genes evident among the currently known *Psa* lineages, combined with capacity for lateral transmission, means that new *Psa* variants – either from the source population or produced *de novo* by recombination and horizontal gene transfer – are to be expected. This study has identified 17 effectors conserved across all *Psa* lineages that might usefully be the focus of a breeding program, while metabolic and physiological factors should not be overlooked. In this regard it maybe of more than passing interest that the only significant genomic change that has thus far occurred among the extant *Psa*-V lineage involves three ICEs, which despite highly diverse sets of core genes contain an identical cargo of accessory genes predicted to manipulate host metabolism.

Human activities have long shaped the evolution of microbes [97] Ensuing feedback effects on the evolution of human populations – particularly in the context of human disease – are widely recognized [71], [98], [99]. Less well understood are anthropomorphic impacts on the evolution of plant pathogens [3]. Although our study provides just the first glimpse of a plant disease as it emerges in the face of domestication, continued analysis of the population processes and genetic phenomena underpinning evolution of *Psa* stand to enlighten both critical events driving the evolution of virulence and broader issues associated with the evolutionary interaction between microbes, their hosts, and the communities of which they are part.

## Supporting Information

Dataset S1 **Artemis input file for depicting positions of SNPs.** Read the readme file. Artemis is required to view the files: http://www.sanger.ac.uk/resources/software/artemis/.
(ZIP)

Figure S1 **Inoculation and sampling areas for pathogenicity assay.** A sample image displaying stab inoculation on kiwifruit plantlets (A). A 2 µL drop of inoculum was suspended on the wound site created with a needle dipped in inoculum. Leaf tissue samples for the quantification of bacterial density were taken as shown in B from the base (A), middle (B), tip (C) and periphery (D).
(TIF)

Figure S2 **Pulsed-field gel electrophoresis profiles of** ***Psa*** **NZ V-13 and J-35 plasmids.** Lanes 3 to 6 display *Psa* V-13 (32 µg) plasmid digested with the restriction enzymes *Bam*H1 (lane 3), *Hin*dIII (lane 4), *Eco*RI (lane 5) and *Not*I (lane 6). *Psa* J-35 (19 µg) plasmid digested with *Not*I is shown in lane 7. Kb+, Hyperladder I and Midrange II ladders are shown in lanes 1, 9 and 10. Gel electrophoresis conditions are stipulated in the methods section.
(TIF)

Figure S3 **Paired-end read depth and coverage of the** ***Psa*** **NZ V-13 chromosome, plasmid and Pacific Island.** Hagfish plot showing the read depth across the *Psa* NZ V-13 chromosome and plasmid (A) and the Pacific Island (B).
(TIF)

Figure S4 **Analysis of the location of the Pacific and Mediterranean islands in** ***Psa*** **NZ V-13 and** ***Psa*** **I-12.** (A) Analysis of the insertion site of the ICEs from *Psa* NZ V-13 and *Psa* I-12, and the ability of these elements to excise and circularise *in vitro*. PCR was carried out using primer sets designed to detect circularisation (lanes 2, 8, 15 and 21), excision (lanes 3, 9, 16 and 22) and to identify the *att* site the ICE was inserted into (lanes 4–7, 10–13, 17–20 and 23–26). Primer combinations and sequences are shown in (B) and (C) respectively.
(TIF)

Figure S5 **Type 3 secreted effector repertoires of** ***Psa*** **strains.** T3SE presence (black), absence (white) or presence with disruption, truncation or incomplete sequence (grey) is displayed for all sequenced genomes. Strains are colored according to their phylogenetic classification (Figure 2A). T3SE presence on a predicted genomic island or transposon is indicated in the bottom row. The exchangeable effector locus is designated in yellow.
(TIF)

Figure S6 **Phylogeny of HopZ effectors.** Phylogeny constructed using the Geneious tree builder based on HopZ protein alignments generated by ClustalW.
(TIF)

Figure S7 **Pathogenicity assay of** ***Psa*** **and** ***Pmp*** **strains on kiwifruit.** The growth of the canker-causing *Psa* J-35 (blue), NZ V-13 (red), and K-26 (purple) isolates was assayed on the ‘Hort16A’ (A) and ‘Hayward’ (B) cultivars of kiwifruit, along with the low-virulent NZ LV-5 (green) and a strain of *P. syringae* pv. *morsprunorum* (*Pmp*, yellow) that causes canker disease in *Prunus* spp. The average bacterial density (cfu ± SE) was assayed in the stem tissue at day 0 immediately following stab inoculation, as well as in the base of the first leaf above the inoculation site (no *Psa* or *Pmp* observed, data not shown). The bacterial density was quantified in the base of the first leaf above the inoculation site (dark colored bar), the center of the leaf along the mid-vein (medium colored bar), and at the leaf tip and periphery (light colored bar) 4, 8 and 14 days after inoculation.
(TIF)

Protocol S1 **AWK-script findUniqueBlast.awk.**
(ZIP)

Table S1 **Comparison of** ***Psa*** **with other completed** ***P. syringae*** **genomes.**
(DOCX)

Table S2 **Core** ***Psa*** **genes under positive selection.**
(DOCX)

Table S3 **Outbreak clade-specific genes.**
(DOCX)

Table S4 **Comparison of the effector complement at the** ***Psa*** **EEL locus.**
(DOCX)

Table S5 **Pathogenicity assay analysis of variance.**
(DOCX)
