# Correction: Genomic Analysis of the Kiwifruit Pathogen *Pseudomonas syringae* pv. *actinidiae* Provides Insight into the Origins of an Emergent Plant Disease

**DOI:** 10.1371/annotation/af157ddc-200a-4105-b243-3f01251cc677

**Published:** 2013-09-10

**Authors:** Honour C. McCann, Erik H. A. Rikkerink, Frederic Bertels, Mark Fiers, Ashley Lu, Jonathan Rees-George, Mark T. Andersen, Andrew P. Gleave, Bernhard Haubold, Mark W. Wohlers, David S. Guttman, Pauline W. Wang, Christina Straub, Joel L. Vanneste, Paul B. Rainey, Matthew D. Templeton

The third to last author's name was spelled incorrectly. The correct name is: Joel L. Vanneste.

