# Supplementary material for: Genomic Analysis of the Kiwifruit Pathogen Pseudomonas syringae pv. actinidiae Provides Insight into the Origins of an Emergent Plant Disease
Source: PLoS Pathog. 2013 Jul 25;9(7):e1003503. doi: 10.1371/journal.ppat.1003503 (PMC3723570; doi:10.1371/journal.ppat.1003503)
Supplement: Dataset S1 — Artemis input file for depicting positions of SNPs. Read the readme file. Artemis is required to view the files: http://www.sanger.ac.uk/resources/software/artemis/. (ZIP) [file ppat.1003503.s001.zip › recomb_snp_Data/ReadMe.rtf]

First, load the Psa_NZ_13V_version_9 genome (fasta file) into Artemis with File>OpenFiles labelled:Pan_J-31details_Psa_V13_assembly_2_annot_2.graphsimple.artPan_K–26details_Psa_V13_assembly_2_annot_2.graphsimple.artcontain the position of SNPs when aligned against the Pan_NZ_13V _version_9 genome.  To view these data the above two files should be loaded into Artemis after firstly loading the NZ13V version 9 genome.  Go to Graph->User Plot and then select the above files (one at a time). The number of SNPs is represented in the form of a frequencyplot (keep the window size at 1).Files labelled:polymorphisms_J_K.fragsGI.tabpolymorphisms_J_K.fragsG0.tabidentify the gene coversion events identified by GENECONV that arise from recombinational events between pairs of aligned strains, and recombinational events from strains outside the three compared, respectively.  These files can be read into Artemis on top of the NZ13V genome.Go to File–>Read An Entry and select the above files (one at a time).Files labelled: coverage_J.txt.tab coverage_K.txt.tabShow the regions not covered by reads from either J–31 or K–26.  These are read into Artemis on top of the NZ13V genome.Go to File–>Read An Entry and select the above files (one at a time).
