# Supplementary figures and images for: Genomic Analysis of the Kiwifruit Pathogen Pseudomonas syringae pv. actinidiae Provides Insight into the Origins of an Emergent Plant Disease
Source: PLoS Pathog. 2013 Jul 25;9(7):e1003503. doi: 10.1371/journal.ppat.1003503 (PMC3723570; doi:10.1371/journal.ppat.1003503)

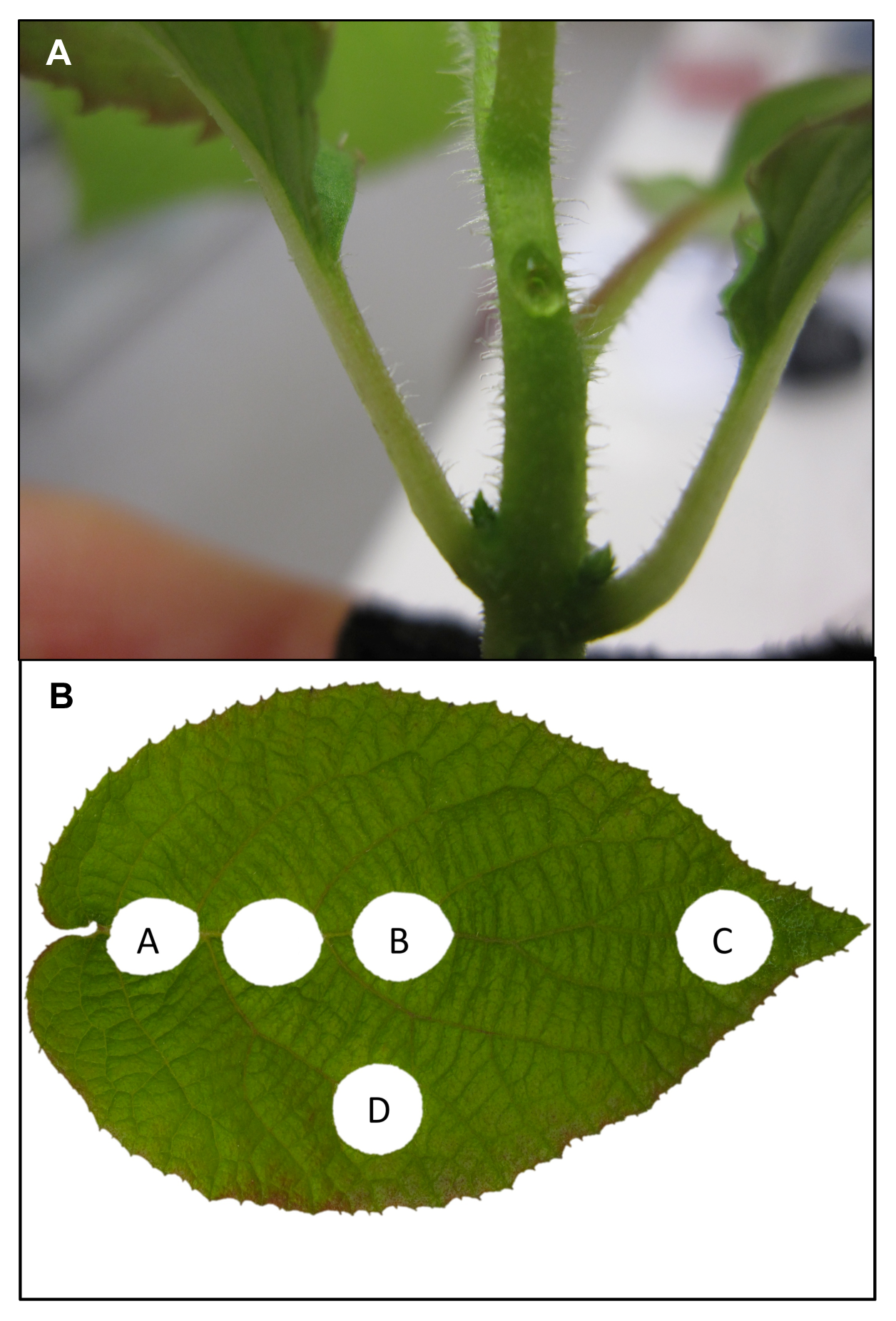

Supplement: Figure S1 — Inoculation and sampling areas for pathogenicity assay. A sample image displaying stab inoculation on kiwifruit plantlets (A). A 2 µL drop of inoculum was suspended on the wound site created with a needle dipped in inoculum. Leaf tissue samples for the quantification of bacterial density were taken as shown in B from the base (A), middle (B), tip (C) and periphery (D). (TIF) [file ppat.1003503.s002.tif]

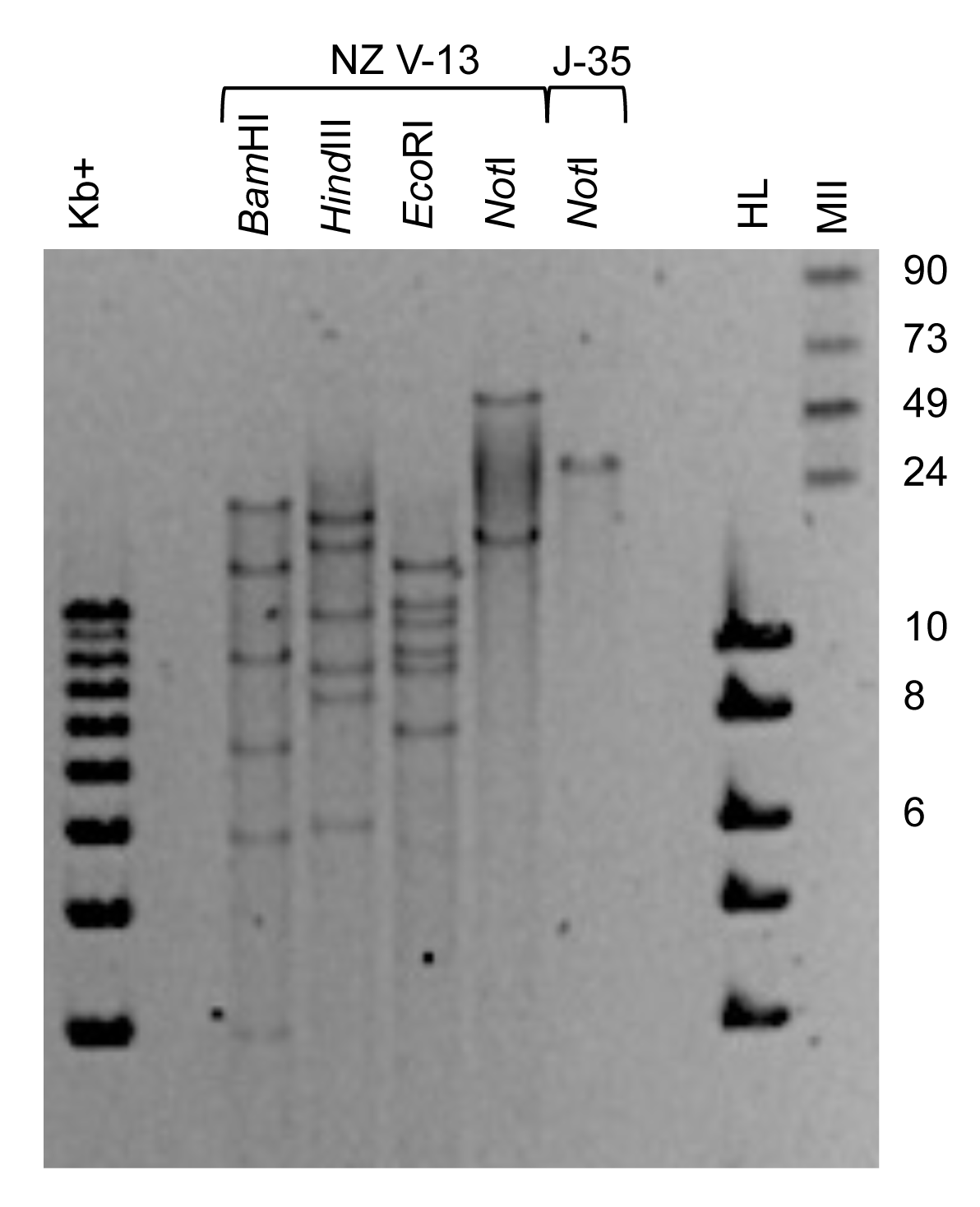

Supplement: Figure S2 — Pulsed-field gel electrophoresis profiles of Psa NZ V-13 and J-35 plasmids. Lanes 3 to 6 display Psa V-13 (32 µg) plasmid digested with the restriction enzymes BamH1 (lane 3), HindIII (lane 4), EcoRI (lane 5) and NotI (lane 6). Psa J-35 (19 µg) plasmid digested with NotI is shown in lane 7. Kb+, Hyperladder I and Midrange II ladders are shown in lanes 1, 9 and 10. Gel electrophoresis conditions are stipulated in the methods section. (TIF) [file ppat.1003503.s003.tif]

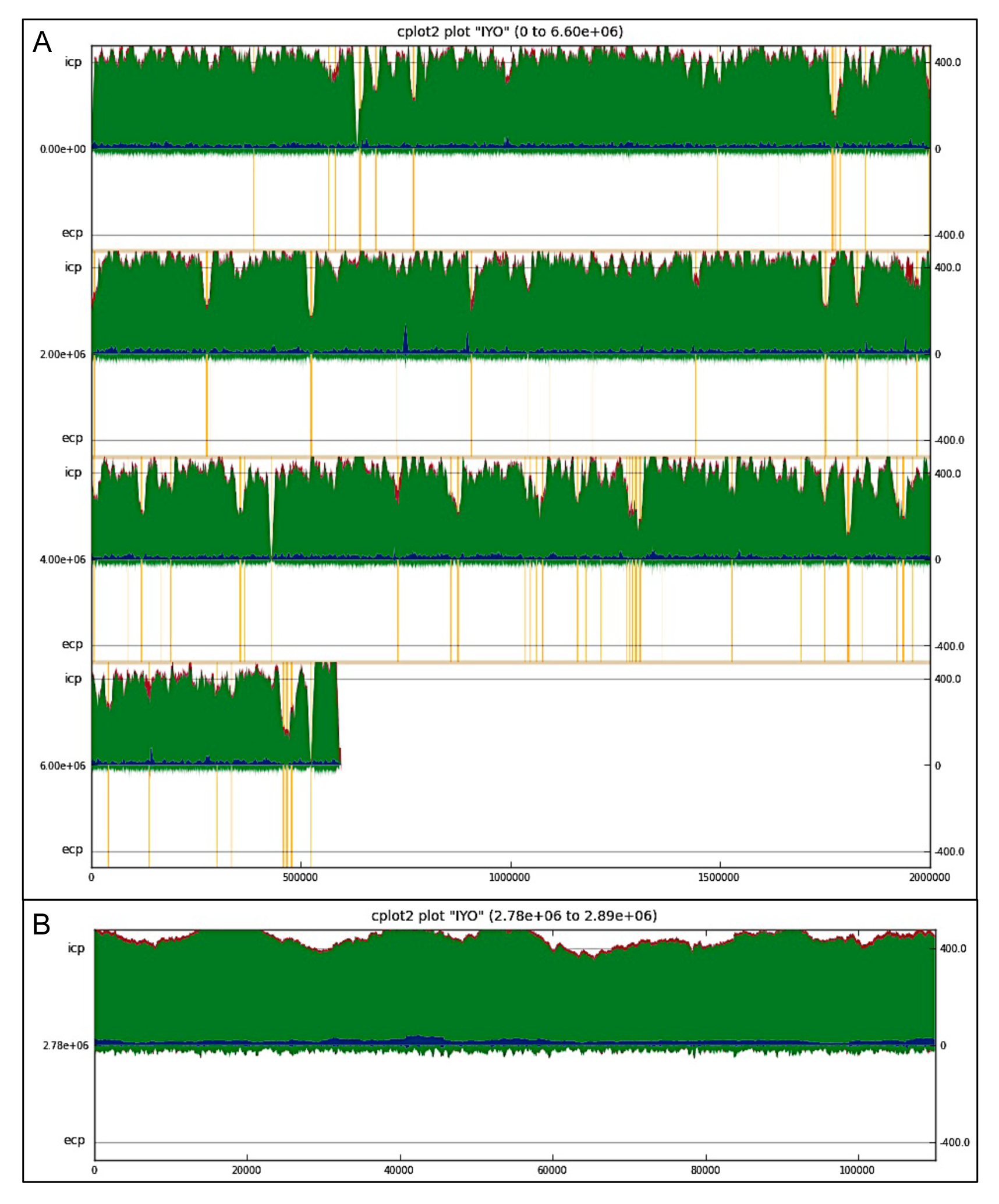

Supplement: Figure S3 — Paired-end read depth and coverage of the Psa NZ V-13 chromosome, plasmid and Pacific Island. Hagfish plot showing the read depth across the Psa NZ V-13 chromosome and plasmid (A) and the Pacific Island (B). (TIF) [file ppat.1003503.s004.tif]

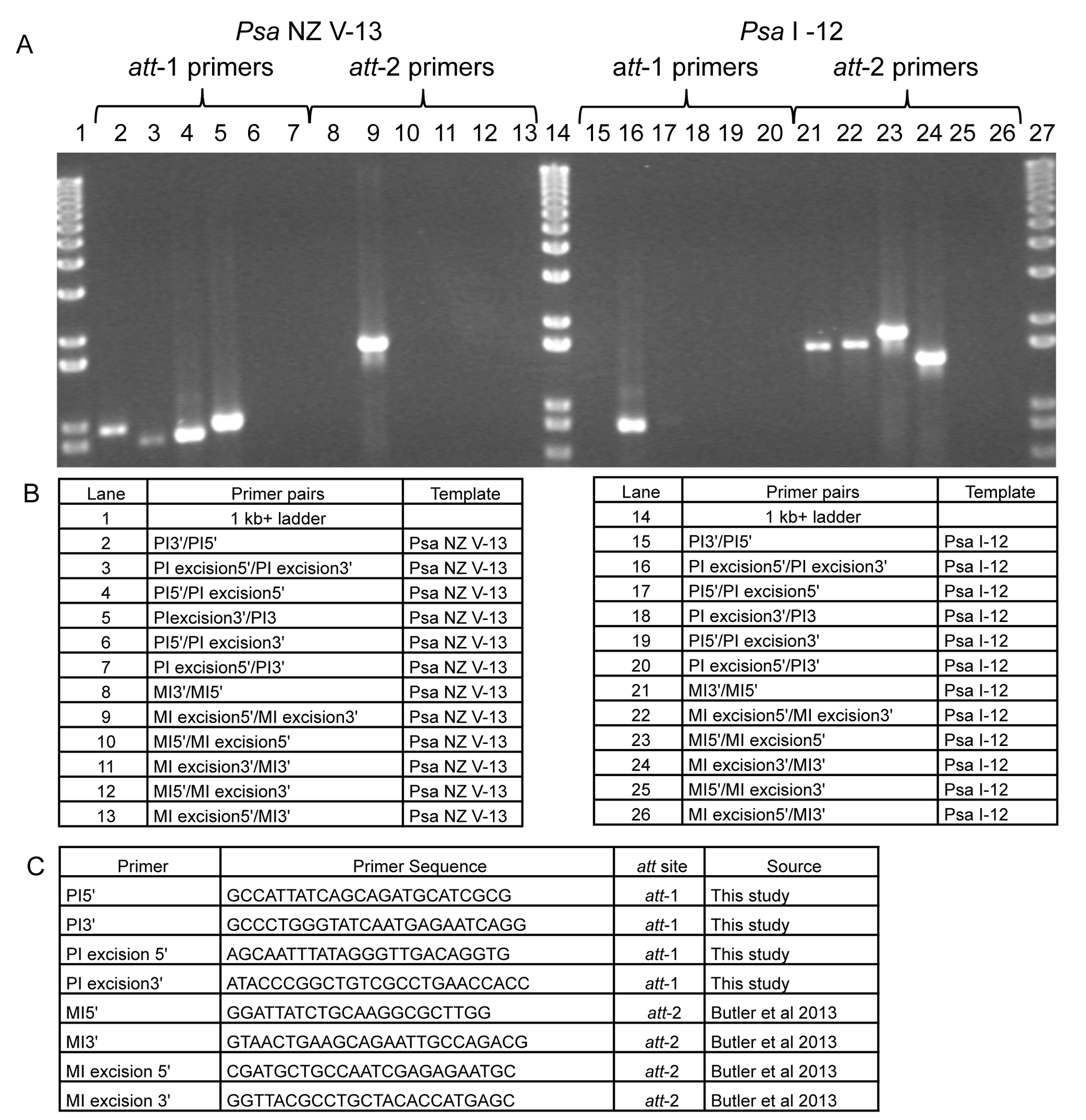

Supplement: Figure S4 — Analysis of the location of the Pacific and Mediterranean islands in Psa NZ V-13 and Psa I-12. (A) Analysis of the insertion site of the ICEs from Psa NZ V-13 and Psa I-12, and the ability of these elements to excise and circularise in vitro. PCR was carried out using primer sets designed to detect circularisation (lanes 2, 8, 15 and 21), excision (lanes 3, 9, 16 and 22) and to identify the att site the ICE was inserted into (lanes 4–7, 10–13, 17–20 and 23–26). Primer combinations and sequences are shown in (B) and (C) respectively. (TIF) [file ppat.1003503.s005.tif]

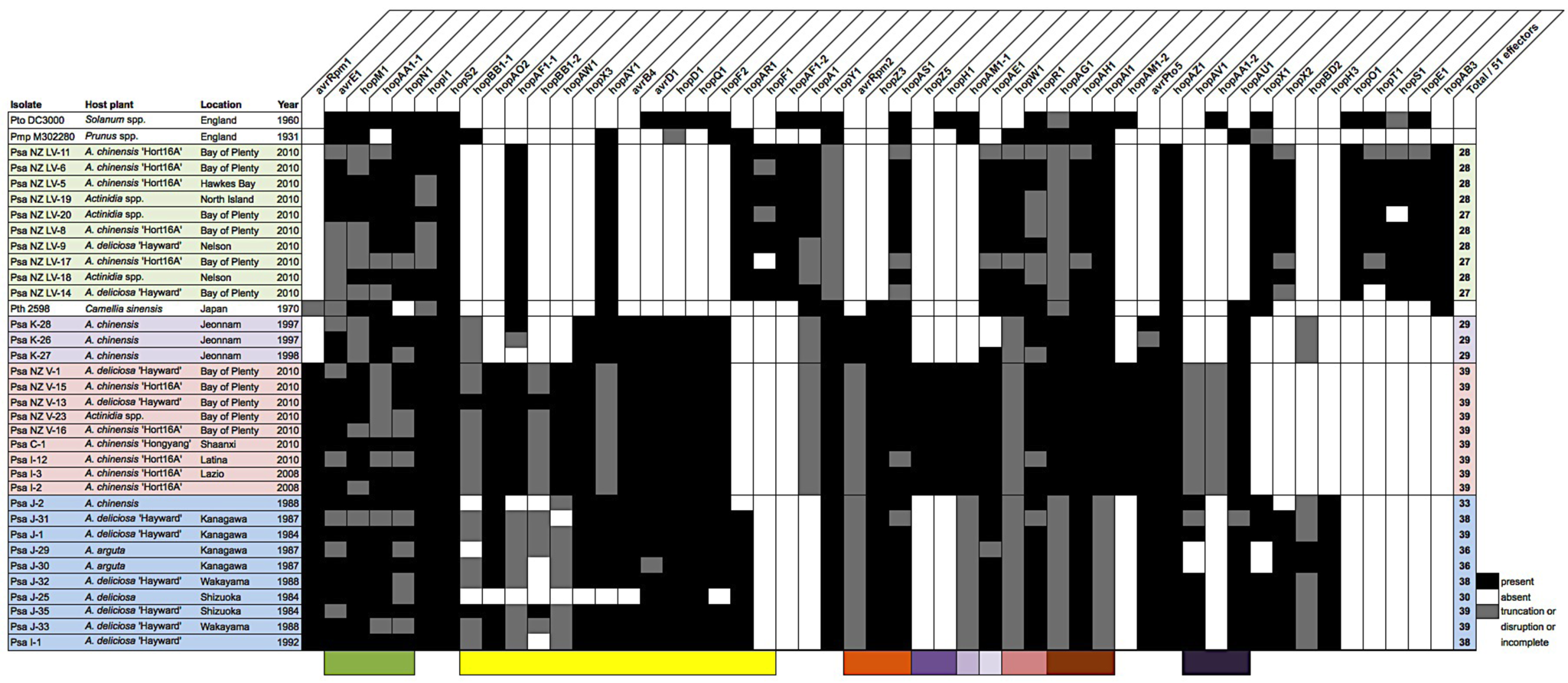

Supplement: Figure S5 — Type 3 secreted effector repertoires of Psa strains. T3SE presence (black), absence (white) or presence with disruption, truncation or incomplete sequence (grey) is displayed for all sequenced genomes. Strains are colored according to their phylogenetic classification (Figure 2A). T3SE presence on a predicted genomic island or transposon is indicated in the bottom row. The exchangeable effector locus is designated in yellow. (TIF) [file ppat.1003503.s006.tif]

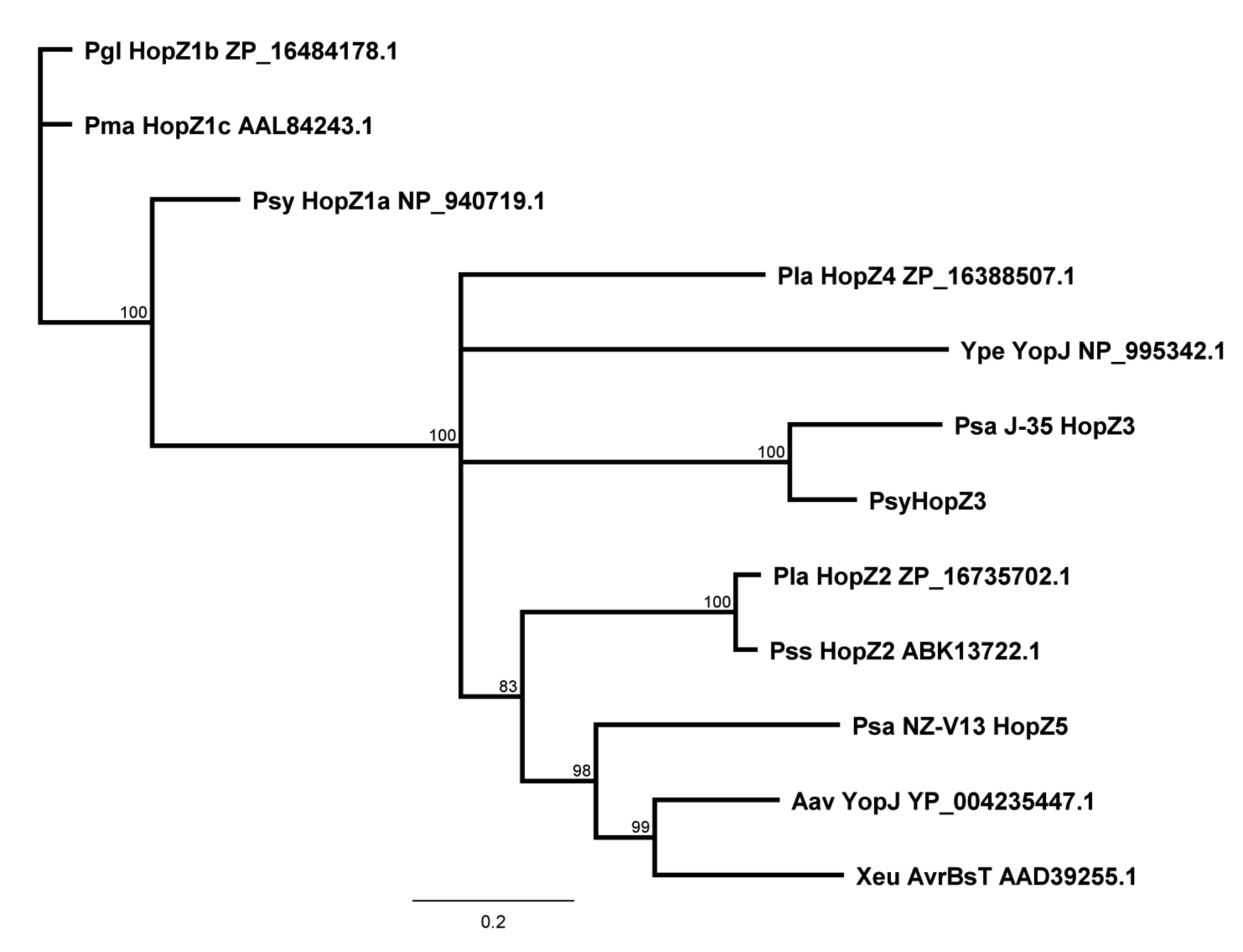

Supplement: Figure S6 — Phylogeny of HopZ effectors. Phylogeny constructed using the Geneious tree builder based on HopZ protein alignments generated by ClustalW. (TIF) [file ppat.1003503.s007.tif]

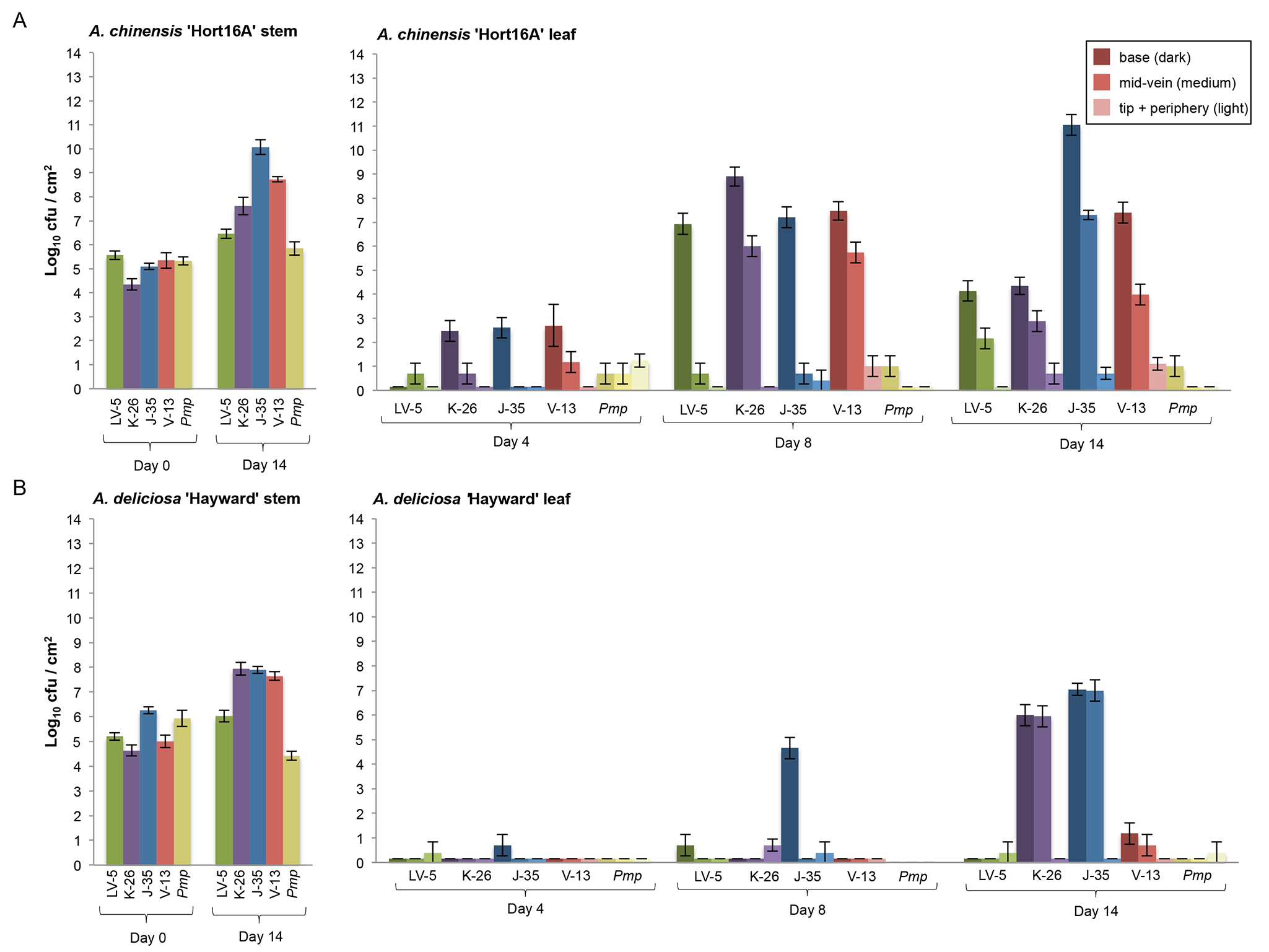

Supplement: Figure S7 — Pathogenicity assay of Psa and Pmp strains on kiwifruit. The growth of the canker-causing Psa J-35 (blue), NZ V-13 (red), and K-26 (purple) isolates was assayed on the ‘Hort16A’ (A) and ‘Hayward’ (B) cultivars of kiwifruit, along with the low-virulent NZ LV-5 (green) and a strain of P. syringae pv. morsprunorum (Pmp, yellow) that causes canker disease in Prunus spp. The average bacterial density (cfu ± SE) was assayed in the stem tissue at day 0 immediately following stab inoculation, as well as in the base of the first leaf above the inoculation site (no Psa or Pmp observed, data not shown). The bacterial density was quantified in the base of the first leaf above the inoculation site (dark colored bar), the center of the leaf along the mid-vein (medium colored bar), and at the leaf tip and periphery (light colored bar) 4, 8 and 14 days after inoculation. (TIF) [file ppat.1003503.s008.tif]
