# Supplementary material for: Genomic Analysis of the Kiwifruit Pathogen Pseudomonas syringae pv. actinidiae Provides Insight into the Origins of an Emergent Plant Disease
Source: PLoS Pathog. 2013 Jul 25;9(7):e1003503. doi: 10.1371/journal.ppat.1003503 (PMC3723570; doi:10.1371/journal.ppat.1003503)
Supplement: Table S1 — Comparison of Psa with other completed P. syringae genomes. (DOCX) [file ppat.1003503.s010.docx]

Table S1. Comparison of *Psa* with other completed *P. syringae* genomes.

| **Strain** | **Chromosome (Mb)** | **ORFs** | **Plasmid (kb)** | **Plasmid ORFs** |
| --- | --- | --- | --- | --- |
| *Pto* DC3000 | 6.40 | 5615 | 74 (A) + 67 (B) | 71 (A) + 77 (B) |
| *Psy* B728A | 6.09 | 5217 | N/A | N/A |
| *Pph* 1448A | 5.93 | 5144 | 132 (A) + 52 (B) | 149 (A) + 60 (B) |
| *Psa* NZ V-13 | 6.50 | 5719 | 71 | 77 |
| *Psa* J-35 | 6.47 | 5282 | 33 | 35 |
