# Supplementary material for: Genomic Analysis of the Kiwifruit Pathogen Pseudomonas syringae pv. actinidiae Provides Insight into the Origins of an Emergent Plant Disease
Source: PLoS Pathog. 2013 Jul 25;9(7):e1003503. doi: 10.1371/journal.ppat.1003503 (PMC3723570; doi:10.1371/journal.ppat.1003503)
Supplement: Table S2 — Core Psa genes under positive selection. (DOCX) [file ppat.1003503.s011.docx]

Table S2. Core *Psa* genes under positive selection.

| **Gene ID**^1^ | **Annotation** | **LRT P**^2^ | **M8 ω^3^** | **BEB PSS**^4^ | **Subcellular localization** |
| --- | --- | --- | --- | --- | --- |
| IYO_24570 | Predicted metal-dependent hydrolase | 3.41E-06 | 999.0 | 2 (0) | Cytoplasmic |
| IYO_26080 | Coenzyme PQQ synthesis protein F | 1.69E-05 | 999.0 | 4 (4) | Periplasmic |
| IYO_04335 | Conserved uncharacterized protein CreA | 1.80E-05 | 999.0 | 3 (2) | Cytoplasmic membrane |
| IYO_15110 | Threonine efflux protein | 3.63E-05 | 999.0 | 3 (2) | Cytoplasmic membrane |
| IYO_08380 | Pantothenate synthetase PanC | 2.49E-04 | 999.0 | 2 (1) | Cytoplasmic |
| **IYO_21565** | Flagellar P-ring protein FlgI | 7.43E-04 | 129.6 | 1 (1) | Periplasmic |
| **IYO_18505** | Manganese transport protein MntH | 1.29E-03 | 777.2 | 2 (0) | Cytoplasmic membrane |
| IYO_22505 | Phosphoglycolate phosphatase | 1.52E-03 | 337.5 | 1 (1) | Cytoplasmic |
| IYO_12100 | Acyl carrier protein AcpP | 2.63E-03 | 999.0 | 1 (0) | Cytoplasmic |
| IYO_09985 | Phospholipase D | 2.65E-03 | 999.0 | 1 (0) | Cytoplasmic membrane |
| **IYO_01585** | Hypothetical protein | 2.89E-03 | 999.0 | 7 (1) | Unknown |
| IYO_03380 | Exodeoxyribonuclease V gamma chain | 3.10E-03 | 67.2 | 1 (0) | Cytoplasmic membrane |
| IYO_19810 | Sarcosine oxidase SolA | 3.19E-03 | 999.0 | 1 (0) | Cell wall |
| IYO_12435 | Soluble lytic murein transglycosylase | 3.20E-03 | 999.0 | 1 (1) | Periplasmic |
| **IYO_12210** | Oxidoreductase | 5.36E-03 | 999.0 | 1 (0) | Cytoplasmic |
| **IYO_09020** | Group 1 glycosyltransferase | 6.14E-03 | 6.1 | 3 (0) | Cytoplasmic |
| IYO_16285 | Outer membrane efflux protein | 9.06E-03 | 999.0 | 1 (1) | Outer membrane |

^1^ Boldface genes have positively selected sites with unique polymorphisms in the outbreak clade

^2^ Probability values for the likelihood ratio test (LRT) for comparisons between M7 and M8

^3^ ω = 999 indicates the rate of synonymous substitution = 0

^4^ Bayes Empirical Bayes determination of positively selected sites (BEB PSS) with posterior probabilities > 0.75; the number of BEB PSS with probabilities > 0.95 shown parenthetically.
