# Supplementary material for: Genomic Analysis of the Kiwifruit Pathogen Pseudomonas syringae pv. actinidiae Provides Insight into the Origins of an Emergent Plant Disease
Source: PLoS Pathog. 2013 Jul 25;9(7):e1003503. doi: 10.1371/journal.ppat.1003503 (PMC3723570; doi:10.1371/journal.ppat.1003503)
Supplement: Table S3 — Outbreak clade-specific genes. (DOCX) [file ppat.1003503.s012.docx]

Table S3. Outbreak clade-specific genes.

| **IYO** | **Description** | **Species/strain (BLAST top hit)** | **Host plant/habitat** |
| --- | --- | --- | --- |
| 00040 | Hypothetical | *Cupriavidus* spp. HMR-1 | Soil? |
| 00045 | Type I restriction enzyme | *Pseudomonas synxantha* BG33R | Insect |
| 00075 | Hypothetical | *Pseudomonas synxantha* BG33R | Insect |
| **00080** | Hypothetical | *Psy* pv. *lachrymans*  M302278 | *Cucumis* spp. (Cucumber) |
| 00100 | Hypothetical | *Pseudomonas s*pp. GM60 | *Populus deltoides* |
| 00105 | Hypothetical | *Marinobacter* spp. BSs20148 | Marine |
| 00110 | Hypothetical | *Marinobacter* spp. BSs20148 | Marine |
| 00115 | Hypothetical | *Pseudomonas stutzeri* ATCC 17588 | Soil (PGPR, Oryza spp.) |
| 00120 | Nucleic acid ATP-dependent helicase | - | No hit |
| 00130 | Type I restriction-modification system | *Pseudomonas stutzeri* ATCC 17588 | Soil (PGPR, Oryza spp.) |
| **00195** | Hypothetical | *Psy* pv. *pisi*  1704B | *Pisum sativum* (Pea) |
| 00200 | ATP-dependent DNA helicase | gamma proteobacterium HdN1 | - |
| 01290 | PDZ/DHR/GLGF domain protein | *Alicycliphilus denitrificans* K601 | Soil |
| 01295 | Hypothetical | *Pseudomonas* spp. Ag1 | Insect (*Anopheles gambiae*) |
| 01305 | Hypothetical | *Psy* pv. *phaseolicola* 1448A | *Phaseolus* spp (Bean) |
| 01320 | Hypothetical | *Psy* pv. *oryzae* 1_6 | Oryza spp. (Rice) |
| 01325 | Hypothetical | *Pseudomonas fluorescens* Q8r1-96 | Soil (PGPR, *Triticum* spp.) |
| 01345 | ATP-dependent endonuclease | *Pseudomonas aeruginosa* C3719 | Human |
| 01565 | Hypothetical | *Yersinia aldovae* ATCC 35236 | Aquatic |
| 01570 | Hypothetical | *Psy* pv. *syringae* 642 | Plant (Nonpathogen) |
| 01575 | Hypothetical | *Psy* pv. *syringae* 642 | Plant (Nonpathogen) |
| 02210 | IS1182 family transposase | *Pseudomonas extremaustralis* 14-3 | Aquatic |
| 02225 | Two component transcriptional regulator | *Sphingomonas* spp. S17 | Stromatolite |
| 03535 | Type III effector HopAW1 | *Psy* pv. *glycinea* B076 | *Glycine* spp. (Soybean) |
| 03590 | Hypothetical | - | No hit |
| 03655 | Hypothetical | *Pseudomonas fluorescens* SS101 | Soil (PGPR, Triticum spp.) |
| 03660 | Hypothetical | *Pseudomonas* spp. TJI-51 | - |
| **05725** | ToxR-activated gene A protein | *Pseudomonas avellanae* BPIC 631 | *Corylus avellana* (Hazelnut) |
| 05735 | Hypothetical | *Psy* pv. *oryzae* 1_6 | *Oryza* spp. (Rice) |
| 07090 | Hypothetical | - | No hit |
| 07840 | Hypothetical | *Psy* pv. *glycinea* race 4 | *Glycine* spp. (Soybean) |
| 07845 | Hypothetical | - | No hit |
| 07850 | Hypothetical | - | No hit |
| 08860 | Hypothetical | - | No hit |
| 08910 | Hypothetical | *Comamonas testosteroni* CNB-2 | Soil |
| 08920 | Hypothetical | *Comamonas testosteroni* CNB-2 | Soil |
| 08930 | Hypothetical | - | No hit |
| **08965** | Prophage CP4-57 regulatory | *Psy* pv. *pisi*  1704B | *Pisum sativum* (Pea) |
| **08970** | Hypothetical | *Psy* pv. *lachrymans* M302278 | *Cucumis* spp. (Cucumber) |
| 11675 | Hypothetical | *Pseudomonas* spp. GM60 | *Populus deltoides* |
| 11680 | Hypothetical | *Pseudomonas* spp. GM60 | *Populus deltoides* |
| 11685 | Hypothetical | *Pseudomonas* spp. GM60 | *Populus deltoides* |
| 11700 | Hypothetical | *Pseudomonas* spp. GM60 | *Populus deltoides* |
| 11705 | Hypothetical | *Pseudomonas* spp. GM60 | *Populus deltoides* |
| 11710 | Hypothetical | *Pseudomonas extremaustralis* 14-3 | Aquatic |
| 11715 | Hypothetical | *Pseudomonas* spp. GM60 | *Populus deltoides* |
| 13555 | Helix-turn-helix domain-containing protein | *Pseudomonas putida* GB-1 | Aquatic |
| 13560 | Hypothetical | *Psy* pv. *oryzae* 1_6 | *Oryza* spp. (Rice) |
| 14025 | Hypothetical | *Pseudomonas* spp. GM79 | *Populus deltoides* |
| 14030 | Hypothetical | *Azoarcus* spp. KH32C | Soil (PGPR) |
| 14050 | Hypothetical | *Psy* pv. *japonica* M301072 | *Hordeum* spp. (Barley) |
| **14485** | Periplasmic substrate-binding protein | *Psy* pv. *morsprunorum* M302280 | *Prunus* spp. (Stone fruit) |
| 14825 | N-acetyltransferase GCN5 | *Pseudomonas* spp. Ag1 | Insect (*Anopheles gambiae*) |
| 14830 | Hypothetical | *Pseudomonas* spp. GM30 | *Populus deltoides* |
| 14835 | Hypothetical | *Pseudomonas viridiflava* UASWS0038 | *Phaseolus* spp (Bean)? |
| 14845 | Hypothetical | No hit | - |
| 14850 | Hypothetical | *Pseudomonas fluorescens* WH6 | Soil (DRB) |
| 14855 | Activator of osmoprotectant transporter | *Pseudomonas putida* ND6 | Aquatic |
| 14865 | Hypothetical | *Rhodobacter sphaeroides* ATCC 17029 | - |
| 14870 | Hypothetical | *Pseudomonas* spp. GM80 | *Populus deltoides* |
| 14875 | Hypothetical | No hit | - |
| 15025 | Nicotine oxidoreductase | *Ktedonobacter racemifer* DSM 44963 | Soil |
| **15595** | Hypothetical | *Xanthomonas axonopodis* pv. *citri* 306 | *Citrus* spp. |
| 15705 | Hypothetical | *Psy* pv. *tomato* NCPPB 1108 | *Solanum* spp. (Tomato) |
| 15710 | Host specificity protein J | *Psy* pv. *tomato* NCPPB 1108 | *Solanum* spp. (Tomato) |
| 15715 | Hypothetical | *Psy* pv. *tomato* Max13 | *Solanum* spp. (Tomato) |
| 15720 | Phage minor tail protein L | *Psy* pv. *tomato* NCPPB 1108 | *Solanum* spp. (Tomato) |
| 15945 | Hypothetical | *Bordetella petrii* DSM 12804 | Aquatic sediment |
| 15950 | Hypothetical | *Variovorax paradoxus* EPS | Soil |
| 15955 | Hypothetical | *Polaromonas naphthalenivorans* CJ2 | Aquatic sediment |
| 16625 | Hypothetical | No hit | - |
| 16630 | PbsX family transcriptional regulator | *Psy* pv. *tomato* DC3000 | *Solanum* spp. (Tomato) |
| 16635 | Hypothetical | *Psy* pv. *oryzae* 1_6 | *Oryza* spp. (Rice) |
| 16640 | Hypothetical | No hit | - |
| 16675 | Hypothetical | *Psy* pv. *glycinea* B076 | *Glycine* spp. (Soybean) |
| 16680 | H-NS family protein MvaT | *Psy* pv. *glycinea* B076 | *Glycine* spp. (Soybean) |
| 16710 | Hypothetical | *Pseudomonas* spp. GM80 | *Populus deltoides* |
| 16715 | Anthranilate synthase component I | *Pseudomonas* spp. GM80 | *Populus deltoides* |
| 16720 | Hypothetical | *Pseudomonas* spp. GM80 | *Populus deltoides* |
| 16735 | Hypothetical | *Vibrio cholerae* RC385 | Marine |
| 16740 | Hypothetical | *Pseudomonas* spp. GM80 | *Populus deltoides* |
| 16765 | Hypothetical | *Psy* pv. *japonica* M301072 | *Hordeum* spp. (Barley) |
| 16770 | Hypothetical | *Psy* pv. *glycinea* B076 | *Glycine* spp. (Soybean) |
| 16775 | Hypothetical | *Psy* pv. *japonica* M301072 | *Hordeum* spp. (Barley) |
| 16780 | Nitroreductase | *Psy* pv. *japonica* M301072 | *Hordeum* spp. (Barley) |
| 16785 | Nitroreductase | *Psy* pv. *japonica* M301072 | *Hordeum* spp. (Barley) |
| 16790 | Hypothetical | *Psy* pv. *japonica* M301072 | *Hordeum* spp. (Barley) |
| 16795 | Hypothetical | *Shewanella baltica* OS185 | Marine |
| 16840 | Hypothetical | *Psy* pv. *glycinea* B076 | *Glycine* spp. (Soybean) |
| 16845 | Hypothetical | *Psy* pv. *glycinea* B076 | *Glycine* spp. (Soybean) |
| 16850 | TniB family protein | *Psy* pv. *glycinea* B076 | *Glycine* spp. (Soybean) |
| 16855 | Integrase catalytic subunit | *Psy* pv. *glycinea* B076 | *Glycine* spp. (Soybean) |
| 16860 | Hypothetical | *Psy* pv. *glycinea* B076 | *Glycine* spp. (Soybean) |
| **16880** | Hypothetical | *Xanthomonas axonopodis* pv. *citri* 306 | *Citrus* spp. |
| **17190** | Hypothetical | *Psy* pv. *aesculi* NCPPB 3681 | *Aesculus* spp. (Horse chestnut) |
| 18255 | Hypothetical | *Psy* pv. *glycinea* B076 | *Glycine* spp. (Soybean) |
| 18275 | Hypothetical | *Psy* pv. *oryzae* 1_6 | *Oryza* spp. (Rice) |
| 18280 | Hypothetical | *Psy* pv. *oryzae* 1_6 | *Oryza* spp. (Rice) |
| 18290 | Putative UV resistance protein | *Psy* pv. *syringae* 642 | Plant (Nonpathogen) |
| 18295 | Hypothetical | *Shewanella baltica* OS678 | Marine |
| 18310 | DMT superfamily transporter | *Halomonas* spp. TD01 | Aquatic |
| 18570 | NERD domain-containing protein | *Pseudomonas chlororaphis* 30-84 | Soil (PGPR, Triticum spp.) |
| 18575 | Hypothetical | - | No hit |
| **18750** | Transposase component | *Psy* pv. *lachrymans* M302278 | *Cucumis* spp. (Cucumber) |
| 22875 | Hypothetical | *Pseudomonas putida* S11 | Soil (PGPR) |
| 22880 | Type III restriction-modification enzyme | *Agrobacterium tumefaciens* F2 | Soil |
| 22885 | Helicase/SNF2 family domain protein | *Psy* pv. *tomato* DC3000 | *Solanum* spp. (Tomato) |
| **26840** | Hypothetical | *Psy* pv. *mori* 301020 | *Morus* spp. (Mulberry) |
| **27125** | Beta-ketoacyl-acyl-carrier synthase I | *Psy* pv. *mori* 301020 | *Morus s*pp. (Mulberry) |
| 27875 | Phage integrase family | *Pseudomonas protegens* Pf-5 | Soil (PGPR) |
| 27880 | Hypothetical | *Psy* pv. *phaseolicola* 1448A | *Phaseolus* spp (Bean) |
| 27895 | Hypothetical | *Psy* pv. *phaseolicola* 1448A | *Phaseolus* spp (Bean) |
| 27905 | Hypothetical | - | No hit |
| 27910 | Hypothetical | *Pseudomonas stutzeri* DSM 4166 | Soil (PGPR, Sorghum spp.) |
| 27915 | Hypothetical | *Psy* pv. *phaseolicola* 1448A | *Phaseolus* spp (Bean) |
| 27920 | NERD domain-containing protein | *Pseudomonas chlororaphis* 30-84 | Soil (PGPR, Triticum spp.) |
| 27930 | Hypothetical | *Pseudomonas aeruginosa* DK2 | Human |
| 27935 | Hypothetical | *Pseudomonas* spp. GM67 | *Populus deltoides* |
| 27940 | Hypothetical | *Pseudomonas aeruginosa* DK2 | Human |
| 27955 | Hypothetical | *Burkholderia* spp. SJ98 | Soil |
| 27990 | TatD-related deoxyribonuclease | *Phenylobacterium zucineum* HLK1 | Human |
| 27995 | Hypothetical | *Pseudomonas aeruginosa* NCMG1179 | Human |
| 28000 | Hypothetical | *Phenylobacterium zucineum* HLK1 | Human |
| 28005 | KAP P-loop | *Pseudomonas aeruginosa* NCMG1179 | Human |
| 28010 | Hypothetical | *Pseudomonas putida* HB3267 | - |
| 28015 | Hypothetical | *Psy* pv. *syringae* 642 | Plant (Nonpathogen) |
| 29160 | Hypothetical | No hit | - |
| **29245** | Hypothetical | *Xanthomonas axonopodis* pv. *citri* 306 | *Citrus* spp. |
| **29325** | Coenzyme F390 synthetase | *Psy* pv. *pisi* 1704B | *Pisum sativum* (Pea) |
| **29330** | GCN5-related N-acetyltransferase | *Psy* pv. *aesculi*  NCPPB 3681 | *Aesculus* spp. (Horse chestnut) |
| **29340** | Multimeric flavodoxin WrbA | *Psy* pv. *aesculi* 0893_23 | *Aesculus* spp. (Horse chestnut) |
| **29345** | Drug/metabolite transporter permease | *Psy* pv. *aesculi* NCPPB 3681 | *Aesculus* spp. (Horse chestnut) |
| 29410 | Hypothetical | No hit | - |
| 29415 | RulB protein | *Psy* pv. *oryzae* 1_6 | *Oryza* spp. (Rice) |
| 29430 | Hypothetical | *Psy* pv. *oryzae*  1_6 | *Oryza* spp. (Rice) |
| 29435 | Hypothetical | *Psy* pv. *oryzae* 1_6 | *Oryza* spp. (Rice) |
| 29465 | Hypothetical | *Psy* pv. *glycinea* B076 | *Glycine* spp. (Soybean) |

Locus tags and annotations refer to orthologs present in *Psa* NZ V-13. Boldface locus tags have top BLAST hits to pathogens of woody plants and/or pathogens exhibiting a vascular infection strategy. PGPR refers to plant growth promoting rhizobacteria, and DRB refers to deleterious rhizobacteria.
