# Supplementary material for: Genomic Analysis of the Kiwifruit Pathogen Pseudomonas syringae pv. actinidiae Provides Insight into the Origins of an Emergent Plant Disease
Source: PLoS Pathog. 2013 Jul 25;9(7):e1003503. doi: 10.1371/journal.ppat.1003503 (PMC3723570; doi:10.1371/journal.ppat.1003503)
Supplement: Table S4 — Comparison of the effector complement at the Psa EEL locus. (DOCX) [file ppat.1003503.s013.docx]

Table S4. Comparison of the effector complement at the *Psa* EEL locus.

| **Strain ID** | **Effectors present in the EEL^1^** |
| --- | --- |
| *Psa* V-13 | *NRPS cluster* hopQ1 hopD1 avrD1 avrB4 hopAY1 hopX3 hopAW1 hopBB1-2 hopAF1-1 hopAO2 hopBB1-1 *hopF2* |
| *Psa* J-25 | *hopQ1 hopD1 avrD1 hopAR1* |
| *Psa* J-35 | hopQ1 hopD1 *arvD1 hopX3 hopAW1* hopAY1 hopAF1-1 hopBB1-2 hopAO2 hopBB1-1 hopF2 *avrB4* *hopAR1* |
| *Psa* K-28 | hopQ1 hopD1 avrD1 *hopF2* avrB4 hopAY1 hopX3 |
| *Psa* K-26 | *hopQ1* hopD1 avrD1 hopF2 *avrB4 hopX3* |
| *Psa* LV-5 | hopAY1 hopAF1-1 hopAR1 hopF1 |
| *Pth* 2598 | *hopAY1* |

^1^ The effector complement in the exchangeable effector locus (EEL) of *Psa* NZ V-13, and hypothesized arrangement of the EEL in other strains with reference to *Psa* NZ V-13. Underlined effectors are located on the same contig or scaffold and are thought to be members of the EEL; italicized effectors are (or may be) outside the EEL, as is the non-ribosomal peptide synthase (NRPS) cluster in *Psa* NZ V-13.
