# Supplementary material for: Genomic Analysis of the Kiwifruit Pathogen Pseudomonas syringae pv. actinidiae Provides Insight into the Origins of an Emergent Plant Disease
Source: PLoS Pathog. 2013 Jul 25;9(7):e1003503. doi: 10.1371/journal.ppat.1003503 (PMC3723570; doi:10.1371/journal.ppat.1003503)
Supplement: Table S5 — Pathogenicity assay analysis of variance. (DOCX) [file ppat.1003503.s014.docx]

Table S5. Pathogenicity assay analysis of variance.

|  | **Hort16A stem** | | | | **Hort16A leaf** | | | |
| --- | --- | --- | --- | --- | --- | --- | --- | --- |
| **Source of variation** | d.f. | M.S. | F | p-value | d.f. | M.S. | F | p-value |
| Between plant |  |  |  |  |  |  |  |  |
| Treatment | 4 | 40.41 | 37.53 | <.001 | 4 | 40.51 | 7.03 | <.001 |
| Day | 1 | 73.35 | 68.13 | <.001 | 2 | 25.74 | 4.47 | 0.017 |
| Treatment x Day | 4 | 49.38 | 45.86 | <.001 | 8 | 17.25 | 2.99 | 0.009 |
| Residual | 30 | 1.077 |  |  | 45 | 5.764 | 1.94 |  |
| Within plant |  |  |  |  |  |  |  |  |
| Area | - | - | - | - | 3 | 69.2 | 23.34 | <.001 |
| Treatment x Area | - | - | - | - | 12 | 10.30 | 3.47 | <.001 |
| Area x Day | - | - | - | - | 6 | 8.37 | 2.82 | 0.013 |
| Treatment x Area x Day | - | - | - | - | 24 | 5.92 | 2 | 0.007 |
| Residual | - | - | - | - | 135 | 2.97 |  |  |

|  | **Hayward stem** | | | | **Hayward leaf** | | | |
| --- | --- | --- | --- | --- | --- | --- | --- | --- |
| **Source of variation** | d.f. | M.S. | F | p-value | d.f. | M.S. | F | p-value |
| Between plant |  |  |  |  |  |  |  |  |
| Treatment | 4 | 23.90 | 29.48 | <.001 | 4 | 8.68 | 4.62 | 0.003 |
| Day | 1 | 52.10 | 64.27 | <.001 | 2 | 2.63 | 1.4 | 0.257 |
| Treatment x Day | 4 | 38.16 | 47.08 | <.001 | 8 | 2.63 | 1.4 | 0.223 |
| Residual | 30 | 0.811 |  |  | 45 | 1.88 | 1.32 |  |
| Within plant |  |  |  |  |  |  |  |  |
| Area | - | - | - | - | 3 | 18.07 | 12.73 | <.001 |
| Treatment x Area | - | - | - | - | 12 | 3.54 | 2.5 | 0.005 |
| Area x Day | - | - | - | - | 6 | 2.73 | 1.92 | 0.082 |
| Treatment x Area x Day | - | - | - | - | 24 | 2.06 | 1.45 | 0.096 |
| Residual | - | - | - | - | 135 | 1.42 |  |  |
